# Combined Large Cell Neuroendocrine Carcinomas of the Lung: Integrative Molecular Analysis Identifies Subtypes with Potential Therapeutic Implications

**DOI:** 10.3390/cancers14194653

**Published:** 2022-09-24

**Authors:** Michele Simbolo, Giovanni Centonze, Luca Giudice, Federica Grillo, Patrick Maisonneuve, Anastasios Gkountakos, Chiara Ciaparrone, Laura Cattaneo, Giovanna Sabella, Rosalba Giugno, Paola Bossi, Paola Spaggiari, Alessandro Del Gobbo, Stefano Ferrero, Luca Mastracci, Alessandra Fabbri, Martina Filugelli, Giovanna Garzone, Natalie Prinzi, Sara Pusceddu, Adele Testi, Valentina Monti, Luigi Rolli, Alessandro Mangogna, Luisa Bercich, Mauro Roberto Benvenuti, Emilio Bria, Sara Pilotto, Alfredo Berruti, Ugo Pastorino, Carlo Capella, Maurizio Infante, Michele Milella, Aldo Scarpa, Massimo Milione

**Affiliations:** 1 Department of Diagnostics and Public Health, Section of Pathology, University of Verona, 37134 Verona, Italy; 2 1st Pathology Division, Department of Pathology and Laboratory Medicine, Fondazione IRCCS Istituto Nazionale dei Tumori, 20133 Milan, Italy; 3 Department of Computer Science, University of Verona, 37134 Verona, Italy; 4 Unit of Pathology, Department of Surgical Sciences and Integrated Diagnostics, University of Genoa and Ospedale Policlinico San Martino, 16132 Genoa, Italy; 5 Division of Epidemiology and Biostatistics, IEO, European Institute of Oncology IRCCS, 20132 Milan, Italy; 6 Pathology Department, Humanitas Clinical and Research Center, Humanitas Milan ENETS Center of Excellence, 20089 Milan, Italy; 7 Division of Pathology, Fondazione IRCCS Ca’ Granda Ospedale Maggiore Policlinico, 20122 Milan, Italy; 8 2nd Pathology Division, Department of Pathology and Laboratory Medicine, Fondazione IRCCS Istituto Nazionale dei Tumori, 20133 Milan, Italy; 9 Medical Oncology Department, Fondazione IRCCS, Istituto Nazionale dei Tumori, 20133 Milan, Italy; 10 Thoracic Surgery Unit, Fondazione IRCCS Istituto Nazionale Tumori, 20133 Milan, Italy; 11 Institute for Maternal and Child Health, IRCCS Burlo Garofalo, 34137 Trieste, Italy; 12 Department of Pathology, ASST Spedali Civili of Brescia, 25123 Brescia, Italy; 13 Thoracic Surgery Unit, Department of Medical and Surgical Specialties Radiological Sciences and Public Health, Medical Oncology, University of Brescia, ASST Spedali Civili of Brescia, 25123 Brescia, Italy; 14 Fondazione Policlinico Universitario A. Gemelli IRCCS, 00168 Rome, Italy; 15 Department of Medicine, Section of Oncology, University of Verona, 37134 Verona, Italy; 16 Medical Oncology Unit, ASST Spedali Civili of Brescia, Department of Medical and Surgical Specialties, Radiological Science and Public Health, University of Brescia, 25123 Brescia, Italy; 17 Department of Medicine and Surgery, University of Insubria, 21100 Varese, Italy; 18 Thoracic Surgery, University and Hospital Trust of Verona, 37134 Verona, Italy

**Keywords:** neuroendocrine carcinoma, combined large cell neuroendocrine carcinoma, next-generation sequencing, transcriptomics

## Abstract

**Simple Summary:**

In this manuscript, we offer an integrated molecular analysis of 44 combined large cell neuroendocrine carcinomas (CoLCNECs) in order to deepen the knowledge about these rare histotypes and to clarify their relationship with lung cancers. In the present state of research, molecular studies are still scant, consisting of small and heterogeneous cohorts, and the genomic landscape is poorly characterized. This study shows that CoLCNECs constitute a standalone group of neuroendocrine neoplasm, with three different molecular profiles, two of which overlap with pure LCNEC or adenocarcinoma. CoLCNECs can be considered an independent histologic category with specific genomic and transcriptomic features, different and therefore not comparable to other lung cancers. Indeed, in addition to a histological re-evaluation of lung cancer classification, our study may help to develop a new diagnostic approach for novel and personalized treatments in CoLCNECs.

**Abstract:**

Background: Combined large cell neuroendocrine carcinoma (CoLCNEC) is given by the association of LCNEC with adeno or squamous or any non-neuroendocrine carcinoma. Molecular bases of CoLCNEC pathogenesis are scant and no standardized therapies are defined. Methods: 44 CoLCNECs: 26 with adenocarcinoma (CoADC), 7 with squamous cell carcinoma (CoSQC), 3 with small cell carcinoma (CoSCLC), 4 with atypical carcinoid (CoAC) and 4 napsin-A positive LCNEC (NapA+), were assessed for alterations in 409 genes and transcriptomic profiling of 20,815 genes. Results: Genes altered included *TP53* (n = 30), *RB1* (n = 14) and *KRAS* (n = 13). Targetable alterations included six *KRAS* G12C mutations and *ALK-EML4* fusion gene. Comparison of CoLCNEC transcriptomes with 86 lung cancers of pure histology (8 AC, 19 ADC, 19 LCNEC, 11 SCLC and 29 SQC) identified CoLCNEC as a separate entity of neuroendocrine tumours with three different molecular profiles, two of which showed a non-neuroendocrine lineage. Hypomethylation, activation of MAPK signalling and association to immunotherapy signature specifically characterized each of three CoLCNEC molecular clusters. Prognostic stratification was also provided. Conclusions: CoLCNECs are an independent histologic category. Our findings support the extension of routine evaluation of *KRAS* mutations, fusion genes and immune-related markers to offer new perspectives in the therapeutic management of CoLCNEC.

## 1. Introduction

Combined large cell neuroendocrine carcinomas (CoLCNEC) account for approximately 20–25% of pulmonary LCNECs [1,2,3].

The current WHO classification of lung neoplasms defines CoLCNECs as the association of an LCNEC with a recognizable component of adenocarcinoma (CoADC), squamous cell carcinoma (CoSQC) or, less commonly, spindle cell or giant cell carcinoma [4]. Combinations of LCNEC with SCLC are also reported but the proportion of large cells should be ≥10% of the tumour to define a combined SCLC/LCNEC [1]. Within the spectrum of CoLCNECs, LCNECs that focally express the exocrine marker napsin-A but lack a distinct adenocarcinoma component should also be considered [3]. The association between LCNEC and atypical carcinoid (AC) has also been reported, suggesting that LCNEC may in some cases be the result of accumulating mutations in a previous AC [5,6].

Molecular studies on this rare entity are scant and consist of small and heterogeneous cohorts. In most studies, only the genomic profile has been investigated, highlighting *TP53* as the most frequently altered gene while data on the frequency of mutations affecting *KRAS* and *RB1* genes are discordant [3,7,8,9]. To date, only two studies have analysed these tumours also at a transcriptomic level but their relationship under the umbrella of lung cancers remains unclear [8,10]. Indeed, knowledge about the molecular pathogenesis of combined neuroendocrine/non-neuroendocrine tumours is largely based on studies suggesting a common genetic origin of the two components, especially when the neuroendocrine one is a poorly differentiated carcinoma [9,11,12]. A recent integrated molecular study showed that the histologically different components of the same tumour shared a similar subclonal architecture, especially at a transcriptomic level [10].

Remarkably a therapeutic strategy directed only to one tumour component could be ineffective. In the era of precision medicine, a detailed molecular characterization could guide the therapeutic approach.

We performed an integrated molecular analysis of a well-characterized CoLCNEC series to deepen the knowledge of these rare forms and clarify their relationship among lung cancers.

## 2. Materials and Methods

### 2.1. Cases

A retrospective series (1988–2018) of 44 surgically-resected primary CoLCNEC was collected from 6 Italian institutions with the approval of the local ethics committees (Supplementary Methods).

In addition, 86 surgically-resected primary lung tumours with pure histological features were retrieved for histotypes-based comparison of expression profiles, including 19 adenocarcinomas (ADC), 19 large cell neuroendocrine carcinoma (LCNEC), 11 small cell lung carcinomas (SCLC), 29 squamous cell carcinomas (SQC), and 8 atypical carcinoids (AC). No patient had received preoperative therapy. All cases were formalin-fixed and paraffin-embedded (FFPE) for histological evaluation. Reclassification according to WHO 2021 criteria [1] and confirmation of histological diagnosis were performed by consensus among pathologists before evaluation of each case. Tumours were staged according to the 8th edition of the TNM classification [13]. Eight non-neoplastic lung tissues served as controls for transcriptomic analysis.

### 2.2. Immunohistochemistry

Monoclonal antibodies and staining steps are listed in Table S1. Neuroendocrine markers synaptophysin (Syn) and chromogranin A (ChgA) were used to confirm the neuroendocrine nature and to properly estimate the LCNEC percentage in each CoLCNEC with a non-neuroendocrine counterpart. Napsin-A (NapA) and TTF-1 were used to identify ADC components, p40 to detect squamous components and OTP for carcinoid components. The tumour proliferative index was evaluated only for the neuroendocrine components by counting the percentage of Ki67-positive cells in areas of strongest nuclear labelling [14]. Immunoreactivity and scores for Somatostatin receptor 2A (SSTR2A) were evaluated using a two-tiered system as suggested by Volante et al.: negative for scores of 0 and 1 and positive for 2 and 3 positive [15].

### 2.3. Mutational and Copy Number Variation Status of 409 Cancer Genes

DNA was obtained from an FFPE tumour using 10 consecutive 4-μm sections and the QIAamp DNA FFPE Tissue Kit (Qiagen, Milan, Italy) and qualified according to Simbolo et al. [16]. The Oncomine Tumor Mutational Load (TML) panel (Thermo Fisher Scientific, Milan, Italy) with a next-generation sequencing assay was used (Supplementary Methods). The assay covers 1.65 Mb including the exons of 409 cancer-related genes.

### 2.4. Tumor Mutational Load and Mutational Signatures

TML and mutational spectrum for each sample were evaluated using the Oncomine TML 5.10 plugin on IonReporter (Thermo Fisher Scientific) (Supplementary Methods).

### 2.5. Fusion Gene Detection

The FusionPlex Solid Tumor Panel (ArcherDX, Boulder, CO, USA) was used to screen for fusions in 57 genes (Supplementary Methods).

### 2.6. Gene Expression Analysis by Next-Generation Sequencing

RNA was prepared using ReliaPrep FFPE Total RNA Miniprep System (Promega, Milan, Italy), quantified using Qubit RNA HS Assay Kit (Thermo Fisher), and qualified using RIN analysis of Agilent RNA 6000 Nano Kit on Agilent 2100 Bioanalyzer (Agilent Technologies). RNA with RIN > 5 and concentration over 10 ng/µL was considered suitable. The Ampliseq Transcriptome Human Gene Expression Kit (Thermo Fisher Scientific, MA, USA) was used to analyse the expression status of 20,815 human RefSeq genes. The expression data analysis was subjected to quality control according to Law et al. [17] (Supplementary Methods).

### 2.7. Gene Set Enrichment Analysis

To determine the biological processes differently enriched among all the clusters, we used the GAGE R package [18] and ssGSEA score [19]. We downloaded c2, c5, c6 and H pathways from MSigDB [19,20] and identified the cluster-specific enriched gene sets using the normalized and batch-corrected count matrix. We assessed the ssGSEA score for each pair of samples and gene sets. We performed a z-score normalization of the pathway scores for each sample (Supplementary Methods). A positive correlation between the sample and the specific pathway is represented by a z-score > 0 in a range from −1 to 1. We considered only the differently related pathways (*p*-value < 0.05 according to Benjamini–Hochberg correction for multiple comparisons). All samples were grouped according to their molecular class.

### 2.8. Statistical Analysis

The associations between clinical, immunophenotypical and molecular features and CoLCNEC groups were assessed using the Fisher exact test or the Kruskal–Wallis test, as appropriate. Correction for multiple comparisons was performed according to Benjamini–Hochberg. Overall survival (OS) was assessed from diagnosis to death or last follow-up by the Kaplan–Meier method. The log-rank test was used to assess the survival difference between patient groups. Cox proportional regression analysis was used to assess the association between clinical-pathological features and OS. Hazard ratios (HR) are presented with a 95% confidence interval (CI).

Data analysis was performed using the R environment for statistical computing and graphics (R Foundation, Vienna, Austria—Version 3.6.2) and MedCalc for Windows version 15.6 (MedCalc Software, Ostend, Belgium). All tests were two-sided and *p*-values < 0.05 were considered significant.

## 3. Results

### 3.1. Clinico-Pathological Features

The clinical-pathological features of the 44 CoLCNEC patients are shown in Table 1 and Table S2. The series comprised 18 (41%) females; the median age was 67 years (range: 48–82). All patients had a smoking history. The LCNEC was combined with adenocarcinoma (CoADC) in 26 cases (59.1%), squamous cell carcinoma (CoSQC) in 7 (15.9%), small cell lung cancer (CoSCLC) in 3 (9.1%), atypical carcinoid (CoAC) in 4 (9.1%) and a napsin-A positive LCNEC (NapA+) in the remaining 4 (6.8%).

### 3.2. Immunohistochemical Features

**Figure 1:**
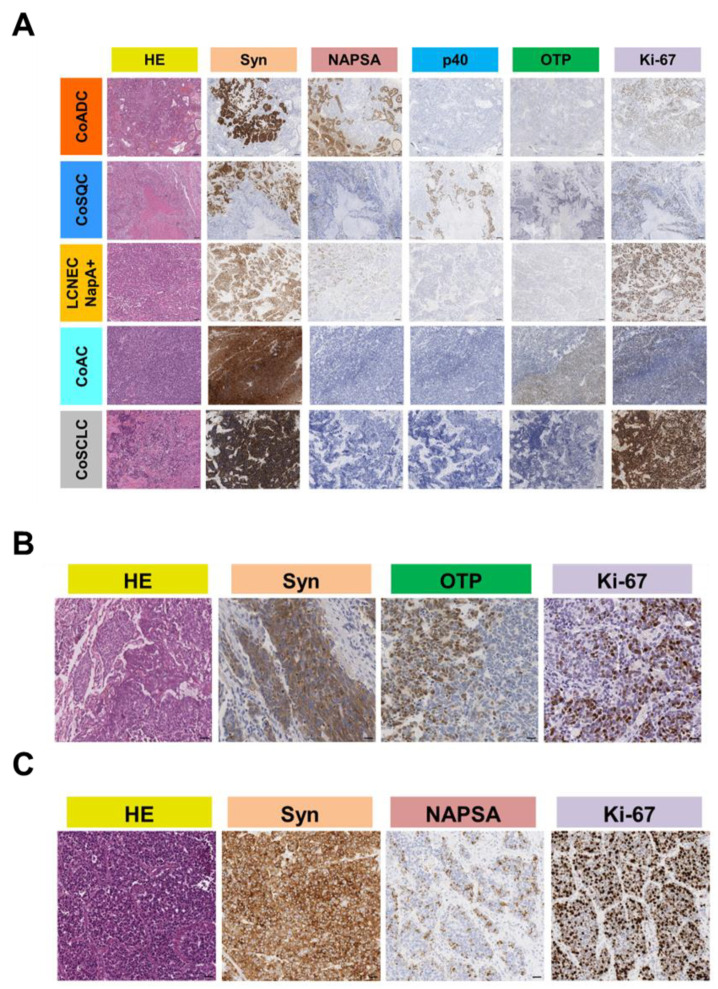
(**A**) Representative cases of combined-LCNEC. Histopathological features of the different components in five selected cases of combined large cell neuroendocrine carcinomas (combined-LCNEC). CoADC, combined-LCNEC with adenocarcinoma; CoSQC, combined-LCNEC with squamous cell carcinoma; LCNEC NapA+, LCNECs showing only immunohistochemical napsin-A positivity but no evidence of a distinct conventional ADC pattern; CoAC combined-LCNEC with atypical carcinoid; CoSCLC, combined-LCNEC with small cell neuroendocrine carcinoma. HE (hematoxylin and eosin); Syn (synaptophysin); NAPSA (napsin-A); p40 (deltaNp63); OTP (orthopedia homeobox protein); Ki-67 (MIB-1); Scale bar = 100 μm. Syn immunostaining identifies the AC, SCLC and LCNEC components, while NAPSA identifies the adenocarcinoma and p40 the squamous component. (**B**) Representative case of CoAC. Ki-67 immunostaining was different between the AC and LCNEC; Scale bar = 50 μm. (**C**) Representative case of LCNEC NapA+. Napsin-A identified exocrine areas; Scale bar = 50 μm.

Immunohistochemical (IHC) data are exemplified in Figure 1A and reported in Table S3. LCNEC, AC and SCLC components were positive for Syn. NapA staining was evident in the glandular component of CoADC and in 4 LCNEC NapA+ (Figure 1B) while p40 was found in the squamous component of CoSQC. OTP expression was observed only in the AC component of 3/4 CoACs cases (*p* = 0.0006; Figure 1C). Positive immunoreactivity for SSTR2A was observed mainly in co-AC (n = 3, 75%), co-SCLC (n = 2, 66.7%) and co-LCNEC Nap + (n = 2, 50%) than co-ADC (n = 5, 19.2%) and co-SQC (n = 1, 14.3%) with a significant difference between groups (*p* = 0.048).

### 3.3. Mutational and Copy Number Status of 409 Genes

All 44 cases were analysed for 409 genes included in the TML assay panel. Sequencing achieved an average coverage of 522 × (119 − 1582×) (Table S4).

**Figure 2:**
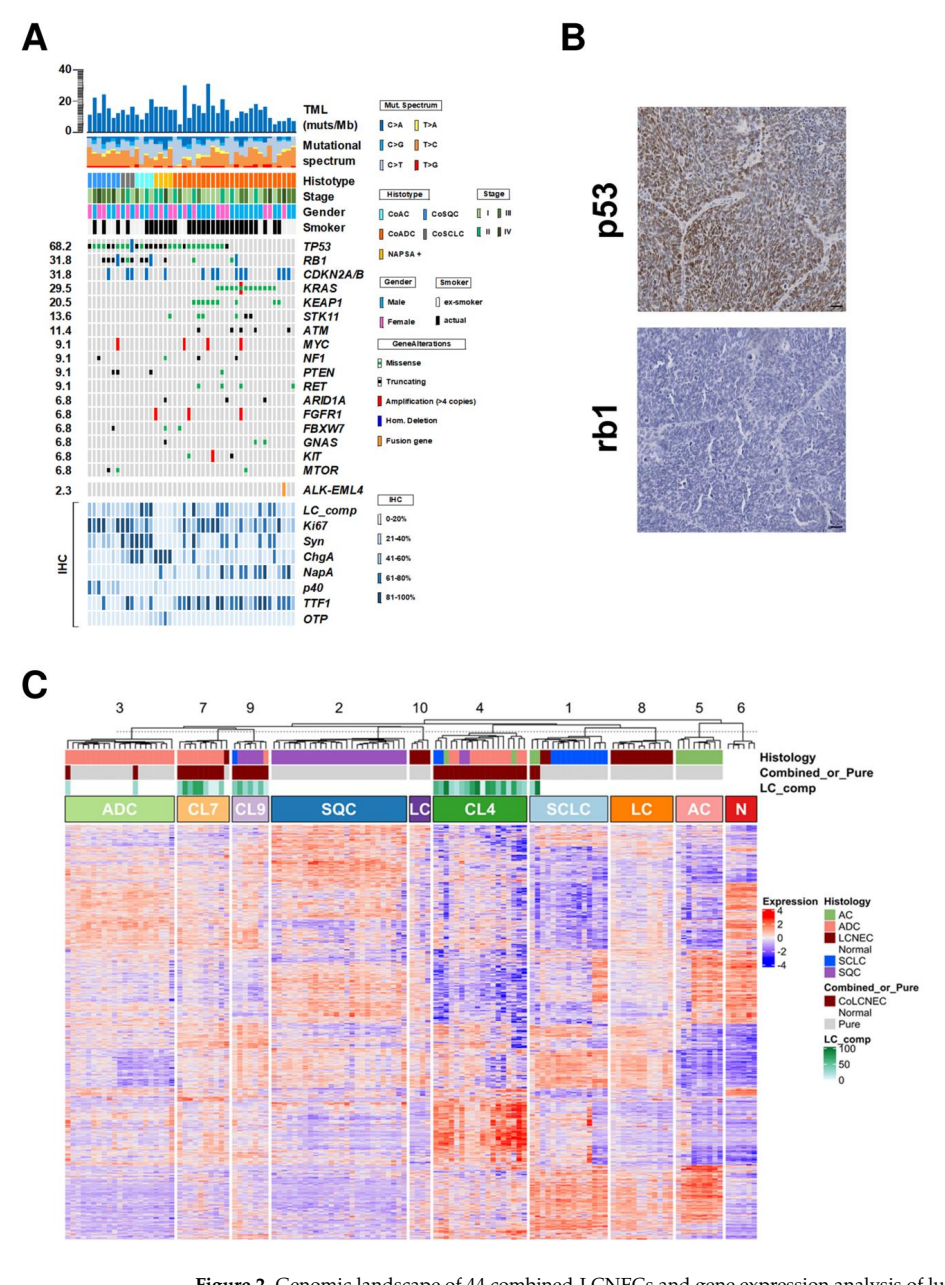
Genomic landscape of 44 combined-LCNECs and gene expression analysis of lung cancer subtypes. (**A**) The upper histogram shows the tumour mutational load. The central matrix shows the 17 genes that were altered at sequencing analysis in at least 3 cases and the fusion transcript identified. Genes are listed according to the frequency of alterations. The bottom matrix shows the proportion of large cell components (LC_comp) and immunopositivity for the indicated markers observed for each sample. ChgA (Chromogranin A); Syn (Synaptophysin); NapA (Napsin-A); p40 (deltaNp63); TTF-1 (thyroid transcription factor 1); OTP (orthopedia homeobox protein). (**B**) Representative case of CoLCNEC affected by alteration in *TP53* and *RB1* genes. Immunostaining for p53 and rb1 protein showed that alterations affected both components; Scale bar = 50 μm. (**C**) Transcriptomic profiles of 38 combined-LCNECs, 86 histologically pure lung cancers [8 atypical carcinoid (AC), 19 adenocarcinoma (ADC), 19 large cell neuroendocrine carcinoma (LCNEC), 11 small cell lung cancer (SCLC), 29 squamous cell carcinomas (SQC)] and 8 non-neoplastic lung samples. The unsupervised hierarchical clustering results are displayed as a heatmap, in which tumour samples are arranged in columns while expression values of 1356 genes identified are arranged in rows; red and blue indicate high and low expression, respectively. The ten clusters identified are labelled with a histotype representative block annotation based on the histological type of samples included and listed as follows: AC, atypical carcinoid; ADC, adenocarcinoma; CL4, combined-LCNEC 4; CL7, combined-LCNEC 7; CL9, combined-LCNEC 9; LC, large cell neuroendocrine carcinoma; N, non-neoplastic lung; SCLC, small cell lung cancer; SQC, squamous cell carcinoma.

Fifty-three genes contained mutations and/or copy number alterations (Table S5). Mutations were 133 (80 missense, 28 nonsense, 13 splice site alterations, 11 frameshifts and 1 frame deletion; Table S6). Mutations were found in at least one gene in all cases and 17 genes were altered in ≥3 cases (Figure 2A; Table 2).

The most frequent alterations involved *TP53* (30/44; 68.2%), followed by *RB1* (14/44; 31.8%) and *KRAS* (13/44; 29.5%). Specifically, *TP53* mutations were enriched in CoAC (4/4; 100%), NapA+ (4/4; 100%), CoSQC (7/7; 100%) and CoSCLC (3/3; 100%) compared with CoADC (12/26; 46.1%) (*p* = 0.005). *RB1* alterations were found mainly in CoSCLC (3/3; 100%), CoAC (3/4; 75%) and CoSQC (4/7; 57.1%) compared with CoADC (3/26; 11.5%) and NapA+ (1/4; 25%) (*p* = 0.0006). Interestingly, *KRAS* mutation was observed only in CoADC, affecting 13/26 (50%) cases including 6 pharmacologically targetable *KRAS G12C* mutations [21] (Table S4).

A validation by IHC for p53 and rb1 was performed and immunostaining for both markers appeared homogeneous and coherent with the mutational pattern (Figure 2B).

Copy number variation status was estimated for all 409 genes by using sequencing data. Focal amplification was observed in 15 genes (Table 2). *MYC* was the most frequently amplified gene (4/44; 9.1%). *CDKN2A/B* locus showed frequent homozygous deletion (14/44; 31.8%) followed by *RB1* (3/44; 6.8%). Based on the chromosomal position of each gene, the status of chromosome arms was defined (Figure S1). Gains in chromosomes 1 were the most frequent alteration observed (25/44; 56.8%) followed by gains in chromosome 8 (28/44; 63.6%) while losses were observed in chromosomes 9 (23/44; 52.3%), 10 (20/44; 45.5%), 13 (24/44; 54.5%) and 15 (19/44; 43.2%).

CoLCNECs had a median TML of 13.2 mutations per Mb (range 4.5–30.2) (Table S7). The mutational signatures did not show any specific pattern.

### 3.4. Fusion Genes

RNA sequencing with the 57-genes panel identified one CoADC with an ALK-EML4 fusion gene, confirmed by FISH analysis that showed positivity in 77% of neoplastic cells in both components (Figure S2).

### 3.5. Gene Expression Profiles

Adequate transcriptome sequencing data were obtained for 38 CoLCNECs and compared with the expression profiles of 86 histologically pure lung cancers and 8 non-neoplastic lung samples (see Materials and Methods).

Unsupervised hierarchical clustering analysis using the Cola package [22] identified 1356 most informative genes (Table S8) and 10 clusters (Figure 2C): one cluster including all non-neoplastic lung samples (N), six clusters each including mainly one single-histology lung cancer subtype (AC, ADC, LC, SCLC and SQC), and three clusters including 34/38 CoLCNECs (CL4, CL7, CL9). The remaining 4 CoLCNECs were 2 CoADCs clustered with the ADC group and 2 CoACs clustering in the SCLC group. The 2 CoADC had >70% of adenocarcinoma component and harboured a *KRAS* mutation in one case and an *ALK-EML4* fusion gene in the other. The 2 CoACs had an LCNEC component of 90% and 30%, respectively, and both cases displayed a *TP53* alteration associated with *RB1* inactivation in one case.

**Figure 3:**
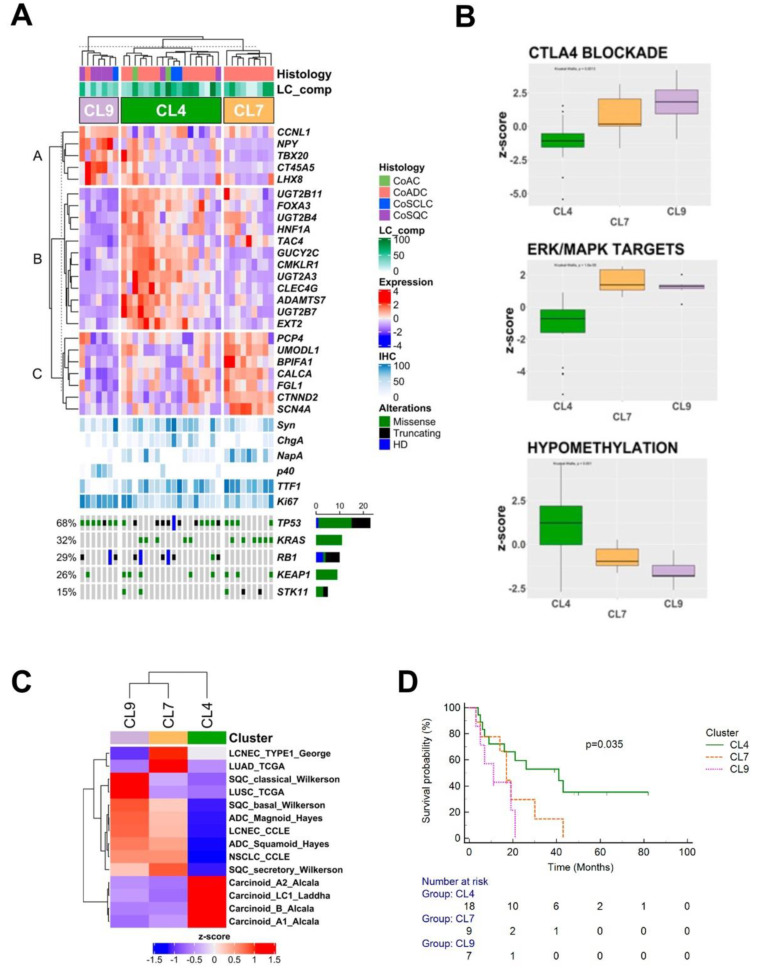
Molecular and clinico-pathological features of combined-LCNEC (CoLCNEC) principal clusters and their contextualization according to current molecular signatures available for lung cancer. (**A**) The upper heatmap shows expression of each characteristic gene included in 24-gene classifier produced following differential expression analysis and comparison of overexpressed genes between clusters. Each tumour sample is arranged in columns, annotated for the histological subtype and grouped according to their expression clustering class (CL4, CL7 and CL9). The central matrix shows immunohistochemical features while the bottom matrix shows selected genomic somatic alterations identified in each sample. (**B**) Box and whisker plots displaying the normalized enrichment z-score for the 3 most representative pathways for each identified molecular class. ssGSEA was used to obtain the enrichment score. (**C**) Comparison between expression profile of each molecular CoLCNEC cluster and currently available signatures of lung cancer. Tumour samples are arranged in columns, grouped according to their expression clustering class (CL4, CL7 and CL9), annotated for the histological subtype. ssGSEA was used to obtain the enrichment score, which represents the degree to which the profiles are similar (red) or different (blue). (**D**) Overall survival significantly divided into 3 major clusters including CoLCNEC. Univariate analysis shows CL9 as the worst performer.

Further characterization of the three CoLCNEC clusters (CL4, CL7, CL9) identified 24 differentially-expressed genes as differentially expressed (Figure 3A) while 141 pathways (Table S9; Figure S3) and 14 lung cancer molecular profiles resulted as differently associated with each of them.

CL4 was the most heterogeneous cluster in terms of LCNEC-associated component (12 CoADCs, 2 CoACs, 2 CoSCLCs and 2 CoSQCs), showed the highest ChgA staining values (*p* = 0.03), and was characterized by recurrent alterations in *TP53* (61.1%), *RB1* (44.4%) and *KRAS* (27.8%) genes. Signatures involving hypomethylation (Figure 3B) were associated with CL4 while molecular profile comparison highlighted a strong similarity to carcinoid tumours, as described elsewhere [23,24] (Figure 3C).

CL7 included only 9 CoADCs most of which harboured mutations in *KRAS* (66.7%) and/or *KEAP1* and *STK11* (33.3% each). Of note, no case displayed *RB1* alterations. GSEA identified a positive correlation between different MAPK-activated (Figure 3B) and NOTCH signalling profiles (Figure S3). This cluster showed a profile quite similar to pure histologically adenocarcinomas (LUAD-TCGA) [25] and to LCNEC type I [8] (Figure 3C).

Finally, CL9 included 5 CoSQCs, 1 CoSCLC and 1 CoADC. This cluster expressed the highest values of Ki-67 and the most frequently altered genes were *TP53* (85.7%) and *RB1* (42.9%). No case had altered *KRAS*. The epithelial–mesenchymal transition, inflammation-related signatures (Figure S3), and the CTLA4 blockade immunotherapy signature (Figure 3B) were correlated to CL9. SQC molecular profiles [26,27] and partially LCNEC profiles described in Cancer Cell Line Encyclopaedia [28] were comparable to this cluster (Figure 3C).

### 3.6. Survival Analysis

The median OS (mOS) was 21 months (95% CI 17–43) in the overall cohort, 39 months for CL4, 17 months for CL7 and 11 for CL9. Patients in CL9 had significantly shorter OS than patients in CL4 (*p* = 0.035; Figure 3D).

Results of Cox proportional regression analysis are shown in Table S10. In univariate analysis, site (central vs. peripheral, *p* = 0.04), Stage (III–IV vs. I–II, *p* = 0.002), Ki-67 (≥55 vs. <55, *p* < 0.0001), NapA (present vs. absent, *p* = 0.01), *ASCL1* (present vs. absent, *p* = 0.02) and cluster (CL9 vs. CL4, *p* = 0.009) were associated with OS. Furthermore, after adjustment for stage and period of diagnosis, patients in CL9 (*p* = 0.01) had significantly poorer survival compared with patients in CL4 (Table S10 and Figure S4).

## 4. Discussion

We performed a genomic and transcriptomic characterization of a cohort of 44 well-characterized CoLCNECs, including 4 CoACs, 26 CoADCs, 4 NapA+, 7 CoSQCs and 3 CoSCLCs. In addition to morphology, immunohistochemistry was crucial for the correct identification of the components, especially for the rare LCNEC NapA+ subtype that shows only immunohistochemical napsin-A positivity but no evidence of a distinct conventional ADC pattern [3].

Genomic analysis showed that *TP53*, *RB1* and *KRAS* genes were frequently altered. A defined distribution of these and other alterations identified in the different histological subtypes of CoLCNEC was reported. Indeed, all CoSCLCs showed simultaneous alteration of *TP53* and *RB1*, while CoADCs showed frequent alterations of *KRAS*, *KEAP1* and *STK11* or the presence of *ALK-EML4* fusion gene in line with the genomic profile of histologically pure SCLC and ADC, respectively. CoSQC, LCNEC NapA+ and CoAC were mainly driven by *TP53* and *RB1* alterations. To date, the genomic profile of CoLCNECs is based on a few studies, in which this rare subtype has been included in cohorts of pure LCNECs [3,7,8,9]. In particular, George et al. [8] reported 8 CoSCLC and 5 CoSQC showing *TP53* and *RB1* alteration in almost all cases. No genomic studies exist on CoACs, while 14 LCNEC NapA+ were recently investigated by Rekhtman et al. [3] showing mutation in *KRAS* and/or *STK11* in 11 cases, similar to our results.

Our study is to our knowledge the first comparing the transcriptomes of CoLCNECs with those of histologically pure lung cancers (AC, ADC, LCNEC, SCLC and SQC). This comparison suggests that CoLCNEC is a standalone entity composed of three distinct clusters: CL4, CL7 and CL9. CL4 showed an expression profile hierarchically linked to neuroendocrine carcinomas (LCNECs and SCLCs), with the greatest number of cases with simultaneous inactivation of *TP53* and *RB1*, and a transcriptomic profile associated with hypomethylation-related signatures. Of interest, none of the markers previously identified as specific to pure histology LCNEC [8] or SCLC [29] (*ASCL1*, *DLL3*, *NOTCHs*, *NEUROD1*, *POU2F3*, *YAP1*) has been identified as differentially expressed in any CoLCNEC cluster while the direct comparison with other lung cancer molecular profiles highlighted a strong similarity to carcinoids described by Alcala et al. [23] and Laddah et al. [24] suggesting a strong neuroendocrine lineage of this cluster. Conversely, CL7 and CL9 transcriptomes were hierarchically closer to non-neuroendocrine lineage. CL7, mainly composed of CoADC showed the greatest number of cases affected by mutation in *KRAS* and/or *STK11*. As expected by such a genomic asset, this cluster showed a transcriptomic profile strongly associated with gene sets linked to the activation of the MAPK pathway, making it a suitable candidate for MEK inhibitor therapy. This is further reinforced by the fact that 3 out of 6 *KRAS* mutations in this cluster were G12C, amenable to target therapy. In this cluster, the non-LCNEC counterpart also influenced the expression profile which resulted in an intermediate between a pure adenocarcinoma and an LCNEC type I [8] described by George and colleagues. Finally, all cases in CL9, which mainly included CoSQC, were driven by *TP53* alterations. The GSEA also showed a relationship between CL9, EMT, inflammation and CTLA4 immunotherapy signature, suggesting that this molecular cluster might be a candidate for immunotherapy. Additionally in this cluster, the non-LCNEC counterpart strongly affected the global molecular lineage of the cluster exhibiting a non-neuroendocrine lineage.

Our study also suggests that these three CoLCNEC clusters have diverse clinical behaviour, with CL4 having a better overall survival with respect to CL9.

## 5. Conclusions

This study shows that CoLCNECs are an independent histologic category within lung cancers with mixed genomic and transcriptomic profiles that carry both prognostic significance and potential therapeutic vulnerabilities.

Our results support the extension of molecular profiling in the diagnostic routine, especially for LCNECs combined with an adenocarcinoma component to evaluate the presence of druggable *KRAS* G12C mutations and fusion genes. Furthermore, transcriptome-based characterization suggests immunotherapy for the worst prognosis molecular group opening up to new therapeutic perspectives.

## Figures and Tables

**Table 1:** Characteristics of the 44 patients with combined-LCNEC of the lung according to their non-LCNEC component.

	All Patients	CoAC	CoADC	NapA+	CoSCLC	CoSQC	*p*-Value *
**Total**	44 (100)	4 (100)	26 (100)	4 (100)	3 (100)	7 (100)	
**Gender**							
Female	18 (40.9)	2 (50.0)	9 (34.6)	2 (50.0)	1 (33.3)	4 (57.1)	
Male	26 (59.1)	2 (50.0)	17 (65.4)	2 (50.0)	2 (66.7)	3 (42.9)	0.79
**Age (Years)**							
Median (range)	67 (43–82)	57 (54–69)	67 (43–77)	58.5 (48–70)	74 (43–78)	71 (47–82)	0.29
**Smoke**							
Actual	29 (65.9)	2 (50.0)	19 (73.1)	4 (100.0)	1 (33.3)	3 (42.9)	
Former	15 (34.1)	2 (50.0)	7 (26.9)	0 (0.0)	2 (66.7)	4 (57.1)	0.17
**Site**							
Central	10 (22.7)	3 (75.0)	4 (15.4)	0 (0.0)	3 (100.0)	0 (0.0)	
Peripheral	34 (77.3)	1 (25.0)	22 (84.6)	4 (100.0)	0 (0.0)	7 (100.0)	**0.0009**
**Stage**							
I	17 (38.6)	2 (50.0)	11 (42.3)	1 (25.0)	1 (33.3)	2 (28.6)	
II	9 (20.5)	1 (25.0)	5 (19.2)	2 (50.0)	1 (33.3)	0 (0.0)	
III	13 (29.5)	1 (25.0)	8 (30.8)	0 (0.0)	1 (33.3)	3 (42.8)	
IV	5 (11.4)	0 (0.0)	2 (7.7)	1 (25.0)	0 (0.0)	2 (28.6)	0.61
**Mitoses (2 mm^2^)**							
Median (range)	25 (10–69)	28.5 (13–43)	24 (10–46)	29.5 (25–44)	27 (21–69)	25 (10–29)	0.51
**Ki-67**							
Median (range)	60 (20–95)	55 (44–75)	59 (27–94)	50 (41–76)	85 (61–91)	68 (20–95)	0.61
**% LCNEC Component**							
Median (range)	40 (10–90)	80 (30–90)	35 (10–90)	75 (30–80)	50 (40–60)	50 (20–60)	0.12
**% Non-LCNEC Component**							
Median (range)	60 (10–90)	20 (10–70)	65 (10–90)	25 (20–70)	50 (40–60)	50 (40–80)	0.12

LCNEC: large cell neuroendocrine carcinoma; CoAC: combined-LCNEC with atypical carcinoid; CoADC: combined-LCNEC with adenocarcinoma; NapA+: LCNEC with sole immunohistochemical expression of napsin-A; CoSCLC: combined-LCNEC with small cell neuroendocrine carcinoma; CoSQC: combined-LCNEC with squamous cell carcinoma. * *p*-value based on the Fisher’s exact for categorical variables and the Kruskal–Wallis test for continuous variables.

**Table 2:** Alteration prevalence for 17 recurrently mutated genes among combined-LCNEC groups according to their non-LCNEC component. Genes are listed according to mutation frequency.

	N. of Patients (%)	CoAC	CoADC	NapA+	CoSCLC	CoSQC	*p*-Value *	Adjusted *p*-Value †
**Total**	44 (100)	4 (100)	26 (100)	4 (100)	3 (100)	7 (100)		
***TP53 ^***	30 (68.2)	4 (100)	12 (46.1)	4 (100)	3 (100)	7 (100)	**0.005**	**0.04**
***RB1 ^***	14 (31.8)	3 (75.0)	3 (11.5)	1 (25.0)	3 (100)	4 (57.1)	**0.0006**	**0.01**
***CDKN2A/B ^***	14 (31.8)	1 (25.0)	9 (34.6)	1 (25.0)	2 (66.7)	1 (14.3)	0.62	0.89
***KRAS***	13 (29.5)	0 (0.0)	13 (50.0)	0 (0.0)	0 (0.0)	0 (0.0)	**0.01**	**0.056**
***KEAP1***	8 (18.2)	0 (0.0)	8 (30.8)	0 (0.0)	0 (0.0)	0 (0.0)	0.26	0.74
***STK11***	6 (13.6)	0 (0.0)	5 (19.2)	1 (25.0)	0 (0.0)	0 (0.0)	0.68	0.89
***ATM***	5 (11.4)	0 (0.0)	5 (19.2)	0 (0.0)	0 (0.0)	0 (0.0)	0.77	0.94
***MYC °***	4 (9.1)	0 (0.0)	3 (11.5)	0 (0.0)	0 (0.0)	1 (14.3)	1.00	1.00
***NF1***	4 (9.1)	0 (0.0)	2 (7.7)	1 (25.0)	0 (0.0)	1 (14.3)	0.60	0.89
***PTEN ^***	4 (9.1)	1 (25.0)	1 (3.8)	0 (0.0)	0 (0.0)	2 (28.6)	0.19	0.74
***RET***	4 (9.1)	0 (0.0)	4 (15.4)	0 (0.0)	0 (0.0)	0 (0.0)	0.86	0.97
***ARID1A***	3 (6.8)	0 (0.0)	2 (7.7)	1 (25.0)	0 (0.0)	0 (0.0)	0.63	0.89
***FGFR1***	3 (6.8)	0 (0.0)	2 (7.7)	1 (25.0)	0 (0.0)	0 (0.0)	0.63	0.89
***FBXW7***	3 (6.8)	0 (0.0)	1 (3.8)	1 (25.0)	0 (0.0)	1 (14.3)	0.36	0.87
***GNAS***	3 (6.8)	0 (0.0)	2 (7.7)	1 (25.0)	0 (0.0)	0 (0.0)	0.63	0.89
***KIT ^***	3 (6.8)	0 (0.0)	3 (11.5)	0 (0.0)	0 (0.0)	0 (0.0)	1.00	1.00
***MTOR***	3 (6.8)	0 (0.0)	1 (3.8)	0 (0.0)	0 (0.0)	2 (28.6)	0.25	0.74

LCNEC: large cell neuroendocrine carcinoma; CoAC: combined-LCNEC with atypical carcinoid; CoADC: combined-LCNEC with adenocarcinoma; NapA+: LCNEC with sole immunohistochemical expression of napsin-A; CoSCLC: combined-LCNEC with small cell neuroendocrine carcinoma; CoSQC: combined-LCNEC with squamous cell carcinoma. * *p*-value based on the Fisher’s exact for categorical variables. † Correction for multiple comparisons according to Benjamini–Hochberg. ^ Mutation and amplification/homozygous deletion were considered. ° Amplification was considered.

## Data Availability

All data generated or analyzed during this study are included in this article and its supplementary material files. Further enquiries can be directed to the corresponding author.

## Supplementary Materials

The following supporting information can be downloaded at: https://www.mdpi.com/article/10.3390/cancers14194653/s1, Figure S1: The panel depicts detail of copy number variation (CNV) in whole chromosomes. The consensus of chromosome CNV was represented in red for copy gain events while blue was used for loss events; Figure S2: *ALK-EML4* fusion gene identification and validation. (A) Schematic diagram of the domains of wild-type *ALK*, wild-type *EML4*, and the identified *ALK-EML4* fusion protein. (B) Representative results of break-apart FISH signals for *ALK* in cancer cells (sample ID 291). Associated red signals (red arrowheads) and green signals (green arrowheads) indicate no split involving the *ALK* gene while lonely red signals (white arrows) indicate genomic rearrangement of *ALK*; Figure S3: Gene set enrichment analysis (GSEA). Heatmaps of: (A) hallmark gene sets from MSigDB collections, (B) oncogenic signature gene sets (C6) and (C) curated gene sets (C2) inferred by gene expression significantly enriched in any of the 3 molecular classes of combined large cell neuroendocrine carcinomas (CoLCNECs). ssGSEA was used to obtain the enrichment score, representing the degree to which the genes in a particular gene set are co-ordinately up- or downregulated; Figure S4: Forest plot of hazard ratios: A forest plot showing the hazard ratio and 95% confidence intervals of Stage, Period of diagnosis and Cluster Table S1: Antibody sources and dilutions; Table S2: Clinico-pathological features of 44 combined large cell neuroendocrine carcinomas (CoLCNECs); Table S3: Immunohistochemical features of 44 combined large cell neuroendocrine carcinomas (CoLCNECs) grouped according to histotype; Table S4: Coverage detail of sequencing analysis performed for each of 44 combined large cell neuroendocrine carcinomas (CoLCNECs); Table S5: Differences in mutation prevalence for 53 mutated genes among co-LCNECs grouped according to histotype. Genes are listed according to their *p*-value; Table S6: Detail of mutations detected in 44 combined large cell neuroendocrine carcinomas (CoLCNECs). Related to Figure 1; Table S7: Tumor mutational load (TML) and signatures of somatic mutations (mutational spectrum) of 44 combined large cell neuroendocrine carcinomas (CoLCNECs); Table S8: List of genes used in unsupervised clustering analysis and differential expression analysis among clusters identified; Table S9: List of gene set included in Hallmark, C2, C5 and C6 MSigDB and GSEA score for each cluster; Table S10: Cox Regression Model for overall survival of 44 combined large cell neuroendocrine carcinomas (CoLCNECs). Refs [17,18,19,20,22,30,31,32,33,34,35,36,37,38,39,40,41,42,43,44,45] have cited in Supplementary Methods.
