# Supplementary material for: Combined Large Cell Neuroendocrine Carcinomas of the Lung: Integrative Molecular Analysis Identifies Subtypes with Potential Therapeutic Implications
Source: Cancers (Basel). 2022 Sep 24;14(19):4653. doi: 10.3390/cancers14194653 (PMC9562868; doi:10.3390/cancers14194653)
Supplement: Supplementary file 1 [file cancers-14-04653-s001.zip › Supplementary Methods.pdf]

Supplementary Methods

3.1. Mutational and copy number variation status of 409 cancer genes

Sequencing was performed on Ion Torrent platform using 20 ng of DNA for each multiplex PCR amplification and subsequent library construction. The quality of the obtained libraries was evaluated by the Agilent 2100 Bioanalyzer on-chip electrophoresis (Agilent Technologies). Emulsion PCR to clonally amplify the libraries was performed with the Ion Chef™ System (Thermo Fisher Scientific, MA, USA). Sequencing was run on the Ion S5XL (Thermo Fisher Scientific, MA, USA) loaded with Ion 540 Chip.

Data analysis, including alignment to the hg19 human reference genome and variant calling, was performed using Torrent Suite Software v.5.10 (Thermo Fisher Scientific, MA, USA). Filtered variants were annotated using a custom pipeline based on vcflib (https://github.com/ekg/vcflib), SnpSift [1], Variant Effect Predictor (VEP) [2] and NCBI RefSeq database. Additionally, alignments were visually verified with the Integrative Genomics Viewer (IGV) v2.8 [3] to further confirm the presence of identified mutations.

3.2. Tumor mutational load and mutational signatures

Copy number variation was evaluated using OncoCNV v6.8 [4]. BAM files obtained by sequencing of tumour samples were compared to BAM files obtained from blood samples. The software includes a multi-factor normalization and annotation technique enabling the detection of large copy number changes from amplicon sequencing data and permits to visualize the output per chromosome.

TML is calculated using a specific algorithm of the Ion Reporter software (Thermo Fisher Scientific, MA, USA) and is expressed as the number of mutations per megabase (muts/Mb), where the number of mutations include nonsynonymous [missense and nonsense single nucleotide variants (SNVs)], plus insertion and deletion variants (InDels) detected per megabase (Mb) of exonic sequences.

The signatures of somatic mutations (mutational spectrum) of individual tumours was obtained considering six major mutation classes: C>T (G:C>A:T); C>A (G:C>T:A); C>G (G:C>C:G); T>A (A:T>T:A); T>C (A:T>G:C); T>G (A:T>C:G) [5,6]. Mutational Signatures in Cancer (MuSiCa) software [7] was used to obtain specific signatures for each sample. Software used .vcf files to align them to the hg19 human reference genome using targeted sequencing parameters. The different types of base-pair substitutions, comprising all nonsynonymous missense and nonsense single nucleotide variants (SNVs), were normalized per megabase (Mb) of exonic sequence. The percentage of each group in each sample was computed.

3.3. Fusion gene detection by Next-Generation Sequencing

The Panel is a targeted sequencing assay that simultaneously detects and identifies fusions in 57 genes without prior knowledge of fusion partners or breakpoints. To prepare the Archer NGS Library, 200 ng of RNA quantified by Qubit RNA HS Assay Kit (Thermo Fisher Scientific, MA, USA), was used in

accordance with Archer FusionPlex Protocol for Ion Torrent. Archer Library preparation reagents included FusionPlex Reagent and panel GSP, and Archer MBC adapters for Ion Torrent. All purifications during library preparation were performed with Agencourt AMPure XP (Beckman Coulter, CA, USA).

Final libraries were quantified with the Ion Library TaqMan Quantitation Kit (Thermo Fisher Scientific, MA, USA) and pooled to equimolar concentration of 40 pM. The prepared library was sequenced using the Ion Chef System (Thermo Fisher Scientific, MA, USA). Sequencing was run on the Ion S5XL (Thermo Fisher Scientific, MA, USA) loaded with Ion 540 Chip. Data analysis was done using ArcherDx Analysis software v5.0.6 using default parameters (ArcherDX).

3.4. Gene expression analysis by Next-Generation Sequencing

We prepared the data composed of 20,815 genes according to Law *et al.* [8], performing a quality control and removing the sample's differences based on how meta information drove the count values. Genes not expressed at a biologically meaningful level in any condition were filtered out to increase the reliability of the mean-variance relationship. In this dataset, the median library size was about 5.8 million, so the filterByExpr function kept genes that had a log-CPM of 1.55 or more in at least three samples. The differences between samples due to the depth of sequencing were then removed and the data was normalised using the trimmed mean of M-values (TMM) [9] method and the count values transformed to log-CPM. We proceeded to check for outsider samples. We assessed how much the single sample medians were distant from the overall one using the z-score. We filtered out 2 samples having a score greater than 3 indicating a distribution of expression values too different from the remaining cases. The final dataset was composed of 128 samples per 18616 genes and was manipulated in two different ways during the downstream analysis. In one case, it was batch corrected, used to find and to pathway-level characterize sample clusters. In the second case, it was not corrected but used directly to get cluster specific enriched genes with the meta information integrated as covariates.

For the first scenario, we treated the dataset following the pipeline included in the package proBatch [10] to check if location, batch and material were biasing the expression values and influencing the grouping of the samples. We performed agglomerative hierarchical clustering Agnes [11,12] to diagnose for batch effects and to evaluate to what extent technical variances still existed in the normalized data. We estimated the method (average, single, complete, ward) which maximized the clustering coefficient, we determined the optimal number of clusters using the average silhouette width and we applied the clustering algorithm. The resulting clusters have been compared using the adjusted Rand index [13] to the samples separated respectively by the batch, material and location information. The batch variable got an index of 0.25 higher than the other information and together with the visualization of the samples by the projection of the gene values on two principal components highlighted that the batch information was driving the grouping of the samples. We then corrected the batch affect using ComBat, described in Johnson *et al.* [14]. The normalized and corrected dataset is used for a semi-supervised consensus

clustering to unveil the similarities inside and between the histological classes. For this operation, we kept only the genes which had not overlapping expression distributions between the histological groups to some extent for modelling a degree of tolerance. The gene distribution composed of its expression values in a group has been summarized with an interval defined by two limits equal to the 0.25 (lower bound) and the 0.75 (upper bound) quantiles. A gene has been selected and defined core for a group when it had a distribution not overlapping with its intervals in the other groups (at least the 60% of the groups). This allowed us to catch the differences and similarities between samples represented by their own almost-core genes without leading the clustering algorithm to provide overfitted results (i.e. clusters perfectly separated due to the starting histological classes). The consensus clustering has been performed with Cola [15]. The method tests six partitioning methods combined to four different feature selection strategies for each possible k number of clusters. After the tests, it provides four scores (Silhouette score, PAC score, Concordance and Jaccard index) that allow to select the best combination. In our case, the method Pam with the standard deviation as approach to retrieve the most informative genes and k equal to ten got the best clustering after that has been tested in 50 runs with resample each time of the 80% of the genes. After having unveiled the real groups of samples, we removed outlier genes for not including them in the downstream analysis. We estimated the coefficients of skewness, kurtosis and linearity together with the position and value of the highest peak in the distributions assumed by each gene in the clusters. We removed the genes that in at least three clusters had a fat-tailed distribution with a kurtosis coefficient greater than 3, a non-linear distribution with a coefficient lower than 1, a peak in the tail above the 0.95 quantile and a peak twice higher the median. These parameters have been found by performing a tuning operation which criteria was to maximize the removal of few manually selected outliers characterized by a low variation in the expression values but a peak in a number of samples lower than the 5% in multiple clusters. Next, we moved to the gene and pathway analysis.

We created a design matrix with the location and material as covariates for each pair of clusters. We computed differential expressed (DE) genes following limma workflow. We intersected the results of all the pairwise comparisons and determined how much a gene was frequently appearing as DE for a specific cluster against every other and poorly appearing in the comparisons not including the group. This step allowed us to get DE cluster-specific genes. We finished the analysis at the gene-level determining the overexpressed genes which were exclusive of one cluster. In other words, the genes which in a group had the distribution described by the 0.3 quantile greater than its 0.8 quantile in the other clusters. We replicated the same step considering the exclusive overexpression of a gene also in a pair of clusters against all the others. In this last case, the distribution of the gene could overlap completely between the clusters of the pair.

Then, we moved to analyse the samples at the pathway-level. We downloaded c6 and H pathways from MSigDB [16,17] and determined the cluster-specific enriched gene sets using the normalized and batch corrected

count matrix. We applied GSEA using GAGE R package [18] between clusters to get pairwise significant up and down regulated pathways. While, we used an approach based on the ssGSEA score [16] for determining the biological processes differently enriched between all the clusters. The ssGSEA score determines how much the genes in a particular set are co-ordinately up- or down-regulated within a specific sample. We assessed the ssGSEA score for each pair of sample and gene set. We represented each pathway in a cluster with the mean score got by its members. We performed a z-score normalization of the pathway scores in the clusters. We ranked the biological processes for each cluster based on the Euclidian distance between its enrichment score and the mean of the other groups. We then selected the top pathways in rank list to characterize the clusters. We downloaded the KEGG signaling pathways [19,20] and performed SPIA [21] to assess which one was perturbed by the set of DE genes which were significant in each designed contrast. All the analysis and results can be reproduced with the R scripts shared at the following github page: https://github.com/LucaGiudice/Supplementary-coLCNEC.

References

1. Cingolani, P.; Patel, V.M.; Coon, M.; Nguyen, T.; Land, S.J.; Ruden, D.M.; Lu, X. Using Drosophila melanogaster as a Model for Genotoxic Chemical Mutational Studies with a New Program, SnpSift. *Frontiers in genetics* **2012**, *3*, 35, doi:10.3389/fgene.2012.00035.
2. McLaren, W.; Pritchard, B.; Rios, D.; Chen, Y.; Flicek, P.; Cunningham, F. Deriving the consequences of genomic variants with the Ensembl API and SNP Effect Predictor. *Bioinformatics* **2010**, *26*, 2069-2070, doi:10.1093/bioinformatics/btq330.
3. Robinson, J.T.; Thorvaldsdottir, H.; Winckler, W.; Guttman, M.; Lander, E.S.; Getz, G.; Mesirov, J.P. Integrative genomics viewer. *Nature biotechnology* **2011**, *29*, 24-26, doi:10.1038/nbt.1754.
4. Boeva, V.; Popova, T.; Lienard, M.; Toffoli, S.; Kamal, M.; Le Tourneau, C.; Gentien, D.; Servant, N.; Gestraud, P.; Rio Frio, T.; et al. Multi-factor data normalization enables the detection of copy number aberrations in amplicon sequencing data. *Bioinformatics* **2014**, *30*, 3443-3450, doi:10.1093/bioinformatics/btu436.
5. Alexandrov, L.B.; Nik-Zainal, S.; Wedge, D.C.; Aparicio, S.A.; Behjati, S.; Biankin, A.V.; Bignell, G.R.; Bolli, N.; Borg, A.; Borresen-Dale, A.L.; et al. Signatures of mutational processes in human cancer. *Nature* **2013**, *500*, 415-421, doi:10.1038/nature12477.
6. Erson-Omay, E.Z.; Caglayan, A.O.; Schultz, N.; Weinhold, N.; Omay, S.B.; Ozduman, K.; Koksal, Y.; Li, J.; Serin Harmanci, A.; Clark, V.; et al. Somatic POLE mutations cause an ultramutated giant cell high-grade glioma subtype with better prognosis. *Neuro Oncol* **2015**, *17*, 1356-1364, doi:10.1093/neuonc/nov027.
7. Diaz-Gay, M.; Vila-Casadesus, M.; Franch-Exposito, S.; Hernandez-Illan, E.; Lozano, J.J.; Castellvi-Bel, S. Mutational Signatures in Cancer (MuSiCa): a web application to implement mutational signatures analysis in cancer samples. *BMC Bioinformatics* **2018**, *19*, 224, doi:10.1186/s12859-018-2234-y.
8. Law, C.W.; Alhamdoosh, M.; Su, S.; Dong, X.; Tian, L.; Smyth, G.K.; Ritchie, M.E. RNA-seq analysis is easy as 1-2-3 with limma, Glimma and edgeR. *F1000Res* **2016**, *5*, doi:10.12688/f1000research.9005.3.
9. Robinson, M.D.; Oshlack, A. A scaling normalization method for differential expression analysis of RNA-seq data. *Genome Biol* **2010**, *11*, R25, doi:10.1186/gb-2010-11-3-r25.
10. Cuklina J, L.C., Willams EG et al. Computational challenges in biomarker discovery from high-throughput proteomic data. *Ph.D. thesis* **2018**.
11. Kaufman L, R.P. *Finding Groups in Data: An Introduction to Cluster Analysis.*; Wiley: New York, 1990.
12. Struyf A, H.M.a.R.P. Integrating Robust Clustering Techniques in S-PLUS. *Computational Statistics and Data Analysis* **1997**.

13. Alexander J, G.a.Y.-Y.A. The impact of random models on clustering similarity. *Journal Machine Learning.* **2017**.
14. Johnson WE, L.C.a.R.A. Adjusting batch effects in microarray expression data using empirical Bayes methods. *Biostatistics* **2007**.
15. Gu Z, S.M., Hübschmann D. cola: an R/Bioconductor package for consensus partitioning through a general framework. *Nucleic Acids Res* **2020**.
16. Subramanian A, T.P., Mootha VK, et al. Gene set enrichment analysis: a knowledge-based approach for interpreting genome-wide expression profiles. . *Proc Natl Acad Sci USA* **2005**.
17. Liberzon A, S.A., Pinchback R, Thorvaldsdóttir H, Tamayo P, Mesirov JP. Molecular signatures database (MSigDB) 3.0. *Bioinformatics* **2011**.
18. Luo W, F.M., Shedden K, Hankenson KD, Woolf PJ. GAGE: generally applicable gene set enrichment for pathway analysis. *BMC Bioinformatics* **2009**.
19. Kanehisa M, G.S. KEGG: kyoto encyclopedia of genes and genomes. *Nucleic Acids Res.* **2000**.
20. Kanehisa M, F.M., Sato Y, Ishiguro-Watanabe M, Tanabe M. KEGG: integrating viruses and cellular organisms. *Nucleic Acids Res.* **2021**.
21. Tarca AL, D.S., Khatri P, et al. A novel signaling pathway impact analysis. *Bioinformatics* **2009**.
