# Supplementary material for: Combined Large Cell Neuroendocrine Carcinomas of the Lung: Integrative Molecular Analysis Identifies Subtypes with Potential Therapeutic Implications
Source: Cancers (Basel). 2022 Sep 24;14(19):4653. doi: 10.3390/cancers14194653 (PMC9562868; doi:10.3390/cancers14194653)
Supplement: Supplementary file 1 [file cancers-14-04653-s001.zip › Table S10.pdf]

Supplementary Table S10. Cox Regression Model for overall survival of 44 combined large cell neuroendocrine carcinomas (co-LCNECs).

Variable		Univariate HR (95% CI)	p-value	Adjusted for Stage and period of diagnosis* HR (95% CI)	p-value
**Combined**					
	Co-ADC	1.00		-	
	co-SQC	1.48 (0.58-3.77)	0.41	-	
	co-AC	1.78 (0.59-5.36)	0.31	-	
	Pure-Nap+	0.53 (0.15-1.83)	0.31	-	
	co-SCLC	3.21 (0.69-14.82)	0.14	-	
**Gender** (Male vs Female)		1.03 (0.52-2.03)	0.94	-	
**Age** (10-year increase)		0.90 (0.65-1.26)	0.55	-	
**Smoke** (Actual vs Ex)		0.68 (0.34-1.39)	0.30	-	
**Pack Years** (Increase of 10 packs)		1.02 (0.86-1.21)	0.79	-	
**Site**					
	Superior Lobe	1.00		-	
	Inferior Lobe	1.34 (0.59-3.04)	0.48	-	
	Hilum region	1.24 (0.36-4.23)	0.73	-	
**Site** (Central vs Peripheral)		2.38 (1.02-5.55)	**0.04**	2.45 (1.05-5.73)	**0.04**
**Stage** (III-IV vs I-II)		2.93 (1.46-5.89)	**0.002**	-	
**Mitoses** (Increase of 10)		0.88 (0.66-1.19)	0.41	-	
**% LCNEC Component** (Increase of 10)		1.00 (0.87-1.15)	0.97	-	
**% Non-LCNEC Component** (Increase of 10)		1.00 (0.87-1.15)	0.97	-	
**Ki67** (>55 vs <55)		6.08 (2.64-14.03)	**<0.0001**	15.55 (5.42-44.6)	**<0.0001**
**Syn** (Increase of 10%)		0.99 (0.88-1.11)	0.84	-	
**ChgA** (Increase of 10%)		0.94 (0.83-1.08)	0.41	-	
**p40** (Present vs Absent)		1.42 (0.58-3.47)	0.44	-	
**TTF1** (Present vs Absent)		0.73 (0.28-1.90)	0.52	-	
**NapA** (Present vs Absent)		0.36 (0.16-0.79)	**0.01**	0.43 (0.19-0.97)	**0.04**
**Alcian** (Present vs Absent)		0.82 (0.41-1.62)	0.56	-	
**CD44** (Present vs Absent)		2.11 (0.84-5.31)	0.11	-	
**OTP** (Present vs Absent)		1.80 (0.54-6.02)	0.34	-	
**SSTR2A** (2--3 vs 0--1)		0.78 (0.38-1.60)	0.50	-	
**SSTR5** (2--3 vs 0--1)		1.18 (0.59-2.36)	0.63	-	
**Rb1**(Present vs Absent)		0.84 (0.40-1.77)	0.64	-	
**p53** (Present vs Absent)		1.67 (0.69-4.07)	0.26	-	
**MDM2** (Present vs Absent)		0.95 (0.44-2.05)	0.90	-	
**MASH1/ASCL1** (Present vs Absent)		0.36 (0.15-0.86)	**0.02**	0.53 (0.21-1.34)	0.18
**Cluster**					
	4	1.00		1.00	
	1	1.52 (0.33-7.12)	0.59	1.51 (0.30-7.65)	0.62
	3	45.92 (6.64-317.42)	**0.0001**	36.51 (4.99-267.3)	**0.0001**
	7	2.43 (0.95-6.19)	0.06	2.81 (0.88-8.95)	0.08
	9	4.38 (1.46-13.21)	**0.009**	4.29 (1.36-13.49)	**0.01**
**RB1** (ALT vs WT)		0.94 (0.46-1.92)	0.86	-	
**TP53** (ALT vs WT)		0.66 (0.33-1.34)	0.25	-	
**KRAS** (ALT vs WT)		1.11 (0.53-2.32)	0.79	-	

**KEAP1** (ALT vs WT)	1.05 (0.43-2.55)	0.92	-
**CDKN2A** (ALT vs WT)	1.64 (0.76-3.53)	0.21	-
**CDKN2B** (ALT vs WT)	1.64 (0.76-3.53)	0.21	-
**STK11** (ALT vs WT)	0.89 (0.31-2.55)	0.83	-
**ATM** (ALT vs WT)	0.73 (0.21-2.47)	0.61	-

* Period of diagnosis categorized in 10 years tiers: Note: Syn, synaptophisin; ChgA, chromogranin A; NapA, napsin A: ALT, altered; WT, wild type; Ki67, Ki67 index; p53, tumor suppressor p53; TTF-1, thyroid transcription factor 1; OTP, orthopedia homeobox protein; SSTR2A, somatostatin receptor 2A; SSTR5, somatostatin receptor 5; mASH1, mammalian achaete scute homolog-1; RB1: retinoblastoma-associated protein; MDM2, mouse double minute 2 homolog.
