# Supplementary material for: Combined Large Cell Neuroendocrine Carcinomas of the Lung: Integrative Molecular Analysis Identifies Subtypes with Potential Therapeutic Implications
Source: Cancers (Basel). 2022 Sep 24;14(19):4653. doi: 10.3390/cancers14194653 (PMC9562868; doi:10.3390/cancers14194653)
Supplement: Supplementary file 1 [file cancers-14-04653-s001.zip › Table S1_R1.pdf]

Supplementary Table S1. Antibody sources and dilutions.

Antigens	Pretratretment	Dilution	Code Number	Clone	Source
Ki67 (M)	High pH 15min - 96°C	1/400	M7240	Mib-1	Dako, Agilent, Denmark
Chromogranin-A (M)	High pH 60min - 98°C	1/100	M0869	Dak-A3	Dako, Agilent, Denmark
Synaptophisin (M)	High pH 15min - 96°C	1/200	M7315	Dak-Synap	Dako, Agilent, Denmark
Napsin A (M)	High pH 30min - 96°C	1/500	NCL-L-Napsin A	IP64	Leica Biosystems
p53 (M)	High pH 30min - 96°C	1/400	M7001	DO-7	Dako, Agilent, Denmark
p40 (M)	High pH 60min - 96°C	1/400	API 3079 G3	BC28	Biocare Medical
TTF-1 (M)	High pH 30min - 96°C	1/2000	M3575	8G7G3	Dako, Agilent, Denmark
SSTR2A (M)	High pH 60min - 98°C	1/800	AB134152	UMB1	Abcam
PD-L1 (M)	Low pH 30min - 96°C	1/50	M3653	22C3	Dako, Agilent, Denmark
OTP (P)	High pH 15min - 96°C	1/400	HPA059342	Polyclonal	Sigma-Aldrich
ASCL1 (M)	High pH 15min - 96°C	1/200	556604	24B72D11.1	BD/Pharmingen San Diego, CA, USA
RB1 PMG (M)	High pH 30min - 96°C	1/200	554136	G3-245	BD/Pharmingen San Diego, CA, USA
Tri-Methyl H3 (M)	Low pH 30min - 96°C	1/400	9733	C36B11	Cell Signaling

Abbreviation: M, monoclonal; Ki67, Ki67 index; p53, tumor suppressor p53; TTF-1, thyroid transcription factor 1; SSTR2A, somatostatin receptor 2A; OTP, orthopedia homeobox protein; ASCL1, achaete scute homolog-1; RB1: retinoblastoma-associated protein.
