# Supplementary material for: Combined Large Cell Neuroendocrine Carcinomas of the Lung: Integrative Molecular Analysis Identifies Subtypes with Potential Therapeutic Implications
Source: Cancers (Basel). 2022 Sep 24;14(19):4653. doi: 10.3390/cancers14194653 (PMC9562868; doi:10.3390/cancers14194653)
Supplement: Supplementary file 1 [file cancers-14-04653-s001.zip › Table S2.pdf]

Supplementary Table S2. Clinico-pathological features of 44 combined large cell neuroendocrine carcinomas (co-LCNECs).

ID	Cluster	Gender	Y	Smoke	Combined Features	LCNEC component %	Combined features component %	Mitosis	ki67%	T	N	M	R	STAGE	Vital Status	FU month	Recurrence	DFS month	Site of Recurrence	Surgery Type
1	NA	M	70	ex smoker	ADC	40	60	19	27,0	2	1	0	0	IIB	dead of desease	190,0	1	181,0	pancreas	Lobectomy
86	NA	F	71	ex smoker	ADC	25	75	46	35,0	2	1	0	0	IIB	dead of desease	180,0	0			Lobectomy
169	CL4	F	55	ex smoker	AC	90	10	43	57,0	1	0	0	0	IA	dead of desease	16,0	0			Lobectomy
208	CL4	M	59	ex smoker	AC	70	30	42	70,0	2	1	0	0	IIB	dead of desease	9,0	1	1,0	liver	Lobectomy
209	CL4	F	74	ex smoker	ADC	70	30	10	27,0	2	0	0	0	IB	alive without disease	63,0	0			Lobectomy
219	CL9	M	82	ex smoker	SQC	50	50	20	68,0	3	1	0	0	IIIA	dead of desease	7,0	0			Lobectomy
238	CL9	F	76	ex smoker	SQC	30	70	29	82,0	1	0	0	0	IA	dead of desease	11,0	0			Partial Resection/Segemental Resection
248	CL4	M	63	ex smoker	ADC	20	80	21	41,0	1	0	0	0	IA	alive without disease	48,0	0			Partial Resection/Segemental Resection
262	CL4	M	63	ex smoker	ADC	40	60	29	41,0	1	0	0	0	IA	dead of desease	39,0	0			Lobectomy
269	CL4	M	71	ex smoker	ADC	30	70	21	94,0	2	2	0	0	IIIA	dead of desease	6,0	0			Lobectomy
279	CL7	M	70	ex smoker	ADC	50	50	35	75,0	4	1	0	0	IIIA	dead of desease	5,0	0			Lobectomy
286	CL3	M	77	ex smoker	ADC	25	75	12	71,0	2	2	0	0	IIIA	dead of desease	4,0	0			Lobectomy
289	CL7	M	45	ex smoker	ADC	30	70	31	38,0	3	1	0	0	IIIA	dead of desease	17,0	1	8,0	lung	Bilobectomy and Pneumectomy
291	CL3	F	44	actual smoker	ADC	30	70	14	36,0	1	2	1	0	IV	alive with disease	29,0	0			Lobectomy
295	CL4	M	57	ex smoker	ADC	25	75	33	91,0	1	X	X	2	IA	alive with disease	17,0	1	1,0	lung	Partial Resection/Segemental Resection
339	CL7	M	74	ex smoker	ADC	40	60	32	76,0	1	0	0	0	IA	dead of desease	43,0	1	42,0	lung	Partial Resection/Segemental Resection
343	CL7	M	73	ex smoker	ADC	30	70	12	48,0	2	0	0	0	IA	dead of desease	19,0	1	17,0	lung	Lobectomy
344	CL9	M	71	ex smoker	ADC	15	85	38	85,0	1	X	0	0	IA	dead of desease	19,0	1	16,0	lung	Partial Resection/Segemental Resection

360	CL7	M	57	ex smoker	ADC	10	90	21	66,0	3	0	0	0	IIB	dead of desease	17,0	0			Lobectomy
364	CL7	M	49	actual smoker	ADC	70	30	30	58,0	2	0	0	0	IB	dead of desease	30,0	1	28,0	cerebellum	Lobectomy
VAL 4	CL7	M	69	actual smoker	ADC	10	90	27	36,0	3	1	0	0	IIIA	alive without disease	17,0	NA			Lobectomy
VAL 5	CL7	F	60	ex smoker	ADC	70	30	24	92,0	1	0	0	0	IA	dead of desease	14,0	NA			Lobectomy
VAL 6	CL7	F	65	ex smoker	ADC	70	30	24	56,0	2	1	0	0	IIB	dead of desease	3,0	NA			Lobectomy
VAL 7	CL4	F	43	ex smoker	SCLC	50	50	69	85,0	3	X	0	0	IIB	alive with disease	14,0	1	2,0	lymph nodes	Partial Resection/Segemental Resection
VAL 8	CL4	M	74	actual smoker	SCLC	60	40	21	61,0	1	0	0	0	IA	dead of desease	7,0	NA			Lobectomy
VAL 17	CL4	F	57	ex smoker	ADC	30	70	18	61,0	3	1	0	0	IIIA	dead of desease	5,0	NA			Lobectomy
VAL 23	CL4	M	70	actual smoker	SQC	50	50	10	30,0	2	2	0	2	IIIA	dead of desease	82,0	NA			Lobectomy
VAL 36	CL4	F	43	actual smoker	ADC	70	30	37	90,0	1	0	0	0	IA	dead of desease	41,0	NA			Partial Resection/Segemental Resection
VAL 37	CL9	M	78	actual smoker	SCLC	40	60	27	91,0	3	1	0	0	IIIA	dead of desease	3,0	NA			Lobectomy
VAL 40	CL9	F	73	actual smoker	SQC	50	50	28	85,0	1	0	0	0	IA	dead of desease	18,0	NA			Lobectomy
VAL 41	CL4	F	59	ex smoker	ADC	90	10	26	42,0	2	0	0	0	IIA	dead of desease	50,0	NA			Lobectomy
VAL 43	CL1	M	54	actual smoker	AC	90	10	15	50,0	1	0	0	0	IA	dead of desease	31,0	NA			Lobectomy
VAL 44	CL4	M	75	ex smoker	ADC	75	25	11	60,0	2	0	0	0	IB	dead of desease	21,0	NA			Lobectomy
VAL 47	CL1	F	69	actual smoker	AC	30	70	13	44,0	2	2	0	0	IIIA	dead of desease	28,0	NA			Partial Resection/Segemental Resection
VAL 48	CL4	M	75	actual smoker	ADC	10	90	31	45,0	3	1	0	0	IIIA	dead of desease	43,0	NA			Bilobectomy and Pneumectomy
VAL 49	CL4	M	77	actual smoker	ADC	40	60	15	45,0	2	2	0	0	IIIA	dead of desease	39,0	NA			Lobectomy
VAL 52	CL9	F	47	actual smoker	SQC	20	80	25	90,0	3	0	1	0	IVA	dead of desease	5,0	NA			Partial Resection/Segemental Resection
VAL 56	CL4	F	58	actual smoker	ADC	70	30	10	50,0	1	0	1	0	IVA	alive with disease	4,0	NA			Bilobectomy and Pneumectomy
VAL 57	CL4	M	71	actual smoker	SQC	40	60	25	51,0	2	1	1	0	IVA	dead of desease	26,0	NA			Lobectomy

VAL 61	CL9	F	64	ex smoker	SQC	60	40	23	95,0	1	2	0	0	IIIA	dead of desease	21,0	NA	Partial Resection/Segemental Resection
28	NA	M	48	actual smoker	NapA+	80	20	NA	45,0	3	0	0		IIB	dead of desease	131,0	NA	
192	NA	F	71	actual smoker	NapA+	70	30	NA	41,0	1	1	0		IIA	alive without disease	120,0	NA	
249	NA	M	55	actual smoker	NapA+	80	20	NA	76,0	2	0	1		IV	dead of desease	6,0	NA	
356	NA	F	64	actual smoker	NapA+	30	70	NA	55,0	2	0	0		IA	dead of desease	53,0	NA	

Note: M, male gender; F, female gender; Y, years; ADC, adenocarcinoma; AC, atypical carcinoid; LCNEC, large cell neuroendocrine carcinoma; NAP+, LCNECs showing only immunohistochemical napsin-A positivity but no evidence of a distinct conventional ADC pattern; SCLC, small cell lung cancer; SQC, squamous cell carcinoma; FU, follow-up; NA, not available.
