# Supplementary material for: Combined Large Cell Neuroendocrine Carcinomas of the Lung: Integrative Molecular Analysis Identifies Subtypes with Potential Therapeutic Implications
Source: Cancers (Basel). 2022 Sep 24;14(19):4653. doi: 10.3390/cancers14194653 (PMC9562868; doi:10.3390/cancers14194653)
Supplement: Supplementary file 1 [file cancers-14-04653-s001.zip › Table S3_R1.pdf]

Supplementary Table S3. Immunohistochemical features of 44 combined large cell neuroendocrine carcinomas (co-LCNECs) grouped according to histotype.

	All patients	CoAC	CoADC	LCNEC NapA +	CoSCLC	CoSQC	p-value*
**Total**	44 (100)	4 (100)	26 (100)	4 (100)	3 (100)	7 (100)	
**Ki67**							
Median [range]	60 [20-95]	55 [44-75]	59 [27-94]	50 [41-76]	85 [61-91]	68 [20-95]	0.61
**Syn**							
Median [range]	50 [10-100]	90 [70-100]	40 [10-90]	100 [100-100]	100 [50-100]	50 [20-60]	**0.0004**
**ChgA**							
Median [range]	22.5 [0-90]	62.5 [10-85]	20 [0-70]	15 [0-70]	50 [50-90]	5 [0-30]	**0.02**
**p40**							
absent	37 (84.1)	4 (100.0)	26 (100.0)	4 (100.0)	3 (100.0)	0 (0.0)	
present	7 (15.9)	0 (0.0)	0 (0.0)	0 (0.0)	0 (0.0)	7 (100.0)	**<0.0001**
**TTF1**							
absent	6 (13.6)	2 (50.0)	0 (0.0)	0 (0.0)	0 (0.0)	4 (57.1)	
present	38 (86.4)	2 (50.0)	26 (100.0)	4 (100.0)	3 (100.0)	3 (42.9)	**0.0005**
**NapA**							
absent	15 (34.1)	4 (100.0)	3 (11.5)	0 (0.0)	3 (100.0)	5 (71.4)	
present	29 (65.9)	0 (0.0)	23 (88.5)	4 (100.0)	0 (0.0)	2 (28.6)	**<0.0001**
**OTP**							
absent	41 (93.2)	1 (25.0)	26 (100.0)	4 (100.0)	3 (100.0)	7 (100.0)	
present	3 (6.8)	3 (75.0)	0 (0.0)	0 (0.0)	0 (0.0)	0 (0.0)	**0.0006**
**SSTR2A**							
absent	31 (70.5)	1 (25.0)	21 (80.8)	2 (50.0)	1 (33.3)	6 (85.7)	
present	13 (29.5)	3 (75.0)	5 (19.2)	2 (50.0)	2 (66.7)	1 (14.3)	**0.048**
**Rb1**							
absent	12 (27.3)	3 (75.0)	2 (7.7)	1 (25.0)	2 (66.7)	4 (57.1)	
present	32 (72.7)	1 (25.0)	24 (92.3)	3 (75.0)	1 (33.3)	3 (42.9)	**0.001**
**p53**							
absent	9 (20.5)	1 (25.0)	3 (11.5)	2 (50.0)	1 (33.3)	2 (28.6)	
present	35 (79.5)	3 (75.0)	23 (88.5)	2 (50.0)	2 (66.7)	5 (71.4)	0.19

**ASCL1**							
absent	7 (15.9)	1 (25.0)	4 (15.4)	0 (0.0)	0 (0.0)	2 (28.6)	
present	37 (84.1)	3 (75.0)	22 (84.6)	4 (100.0)	3 (100.0)	5 (71.4)	0.75

Note:

Syn, synaptophisin; ChgA, chromogranin A; NapA, napsin A; Ki67, Ki67 index; p53, tumor suppressor p53; TTF-1, thyroid transcription factor 1; OTP, orthopedia homeobox protein; SSTR2A: Somatostatin receptors 2A; ASCL1, achaete scute homolog-1; RB1: retinoblastoma-associated protein. CoADC, combined-LCNEC with adenocarcinoma; CoSQC, combined-LCNEC with squamous cell carcinoma; LCNEC NAP+, LCNECs showing only immunohistochemical napsin-A positivity but no evidence of a distinct conventional ADC pattern; CoSCLC, combined-LCNEC with small cell neuroendocrine carcinoma. * p-value based on the Fisher's exact for categorical variables and the Kruskal-Wallis test for continuous variables.
