# Supplementary material for: Combined Large Cell Neuroendocrine Carcinomas of the Lung: Integrative Molecular Analysis Identifies Subtypes with Potential Therapeutic Implications
Source: Cancers (Basel). 2022 Sep 24;14(19):4653. doi: 10.3390/cancers14194653 (PMC9562868; doi:10.3390/cancers14194653)
Supplement: Supplementary file 1 [file cancers-14-04653-s001.zip › Table S4.pdf]

Supplementary Table S4. Coverage detail of sequencing analysis performed for each of 44 combined large cell neuroendocrine carcinomas (co-LCNECs).

ID sample	mean cov T	Combined Features	Cluster
1	280	ADC	NA
86	119	ADC	NA
169	373	AC	CL4
208	637	AC	CL4
209	248	ADC	CL4
219	702	SQC	CL9
238	731	SQC	CL9
248	534	ADC	CL4
262	926	ADC	CL4
269	1582	ADC	CL4
279	792	ADC	CL7
286	767	ADC	CL3
289	950	ADC	CL7
291	1213	ADC	CL3
295	808	ADC	CL4
339	383	ADC	CL7
343	283	ADC	CL7
344	219	ADC	CL9
360	825	ADC	CL7
364	513	ADC	CL7
VAL4	411	ADC	CL7
VAL5	449	ADC	CL7
VAL6	671	ADC	CL7
VAL7	246	SCLC	CL4
VAL8	572	SCLC	CL4
VAL17	219	ADC	CL4
VAL23	260	SQC	CL4
VAL36	308	ADC	CL4
VAL37	865	SCLC	CL9
VAL40	526	SQC	CL9
VAL41	130	ADC	CL4
VAL43	993	AC	CL1
VAL44	231	ADC	CL4
VAL47	279	AC	CL1
VAL48	188	ADC	CL4
VAL49	292	ADC	CL4
VAL52	252	SQC	CL9
VAL56	507	ADC	CL4
VAL57	357	SQC	CL4
VAL61	834	SQC	CL9
28	216	NapA+	NA
192	416	NapA+	NA
249	583	NapA+	NA
356	352	NapA+	NA

Note: ADC, adenocarcinoma; AC, atypical carcinoid; LCNEC, large cell neuroendocrine carcinoma; NAP+, LCNECs showing only immunohistochemical napsin-A positivity but no evidence of a distinct conventional ADC pattern; SCLC, small cell lung cancer; SQC, squamous cell carcinoma; NA, not available.
