# Supplementary material for: Combined Large Cell Neuroendocrine Carcinomas of the Lung: Integrative Molecular Analysis Identifies Subtypes with Potential Therapeutic Implications
Source: Cancers (Basel). 2022 Sep 24;14(19):4653. doi: 10.3390/cancers14194653 (PMC9562868; doi:10.3390/cancers14194653)
Supplement: Supplementary file 1 [file cancers-14-04653-s001.zip › Table S5.pdf]

Supplementary Table S5. Differences in mutation prevalence for 53 mutated genes among co-LCNECs grouped according to histotype. Genes are listed according to their p-value.

Total	All patients	CoAC	CoADC	LCNEC Nap +	CoSCLC	CoSQC	p-value*	p-value†
**Total**	44 (100)	4 (100)	26 (100)	4 (100)	3 (100)	7 (100)		
**Genes**								
**RB1^**	14 (31.8)	3 (75.0)	3 (11.5)	1 (25.0)	3 (100.0)	4 (57.1)	**0.0006**	**0.0324**
**TP53^**	30 (68.2)	4 (100.0)	12 (46.1)	4 (100.0)	3 (100.0)	7 (100.0)	**0.005**	0.13
**KRAS^**	13 (29.5)	0 (0.0)	13 (50.0)	0 (0.0)	0 (0.0)	0 (0.0)	**0.01**	0.18
**ARID2**	1 (2.3)	0 (0.0)	0 (0.0)	0 (0.0)	1 (33.3)	0 (0.0)	0.07	0.47
**CCNE1°**	1 (2.3)	0 (0.0)	0 (0.0)	0 (0.0)	1 (33.3)	0 (0.0)	0.07	0.47
**MSH6**	1 (2.3)	0 (0.0)	0 (0.0)	0 (0.0)	1 (33.3)	0 (0.0)	0.07	0.47
**MYCL1°**	1 (2.3)	0 (0.0)	0 (0.0)	0 (0.0)	1 (33.3)	0 (0.0)	0.07	0.47
**NTRK1**	1 (2.3)	0 (0.0)	0 (0.0)	0 (0.0)	1 (33.3)	0 (0.0)	0.07	0.47
**PTEN^**	4 (9.1)	1 (25.0)	1 (3.8)	0 (0.0)	0 (0.0)	2 (28.6)	0.19	0.87
**MET^**	2 (4.5)	0 (0.0)	1 (3.8)	0 (0.0)	1 (33.3)	0 (0.0)	0.24	0.87
**ERBB4**	1 (2.3)	0 (0.0)	0 (0.0)	1 (25.0)	0 (0.0)	0 (0.0)	0.25	0.87
**MTOR**	3 (6.8)	0 (0.0)	1 (3.8)	0 (0.0)	0 (0.0)	2 (28.6)	0.25	0.87
**NOTCH2^**	1 (2.3)	0 (0.0)	0 (0.0)	1 (25.0)	0 (0.0)	0 (0.0)	0.25	0.87
**SRC°**	1 (2.3)	1 (25.0)	0 (0.0)	0 (0.0)	0 (0.0)	0 (0.0)	0.25	0.87
**KEAP1**	8 (18.2)	0 (0.0)	8 (30.8)	0 (0.0)	0 (0.0)	0 (0.0)	0.26	0.87
**FBXW7**	3 (6.8)	0 (0.0)	1 (3.8)	1 (25.0)	0 (0.0)	1 (14.3)	0.36	0.87
**PTPRD°**	1 (2.3)	0 (0.0)	0 (0.0)	0 (0.0)	0 (0.0)	1 (14.3)	0.40	0.87
**ATRX**	1 (2.3)	0 (0.0)	0 (0.0)	0 (0.0)	0 (0.0)	1 (14.3)	0.41	0.87
**AXL°**	1 (2.3)	0 (0.0)	0 (0.0)	0 (0.0)	0 (0.0)	1 (14.3)	0.41	0.87
**CCND1^**	1 (2.3)	0 (0.0)	0 (0.0)	0 (0.0)	0 (0.0)	1 (14.3)	0.41	0.87
**CD79A°**	1 (2.3)	0 (0.0)	0 (0.0)	0 (0.0)	0 (0.0)	1 (14.3)	0.41	0.87
**KMT2D**	1 (2.3)	0 (0.0)	0 (0.0)	0 (0.0)	0 (0.0)	1 (14.3)	0.41	0.87
**PIK3CA^**	1 (2.3)	0 (0.0)	0 (0.0)	0 (0.0)	0 (0.0)	1 (14.3)	0.41	0.87
**PSIP1**	1 (2.3)	0 (0.0)	0 (0.0)	0 (0.0)	0 (0.0)	1 (14.3)	0.41	0.87
**PTCH1**	1 (2.3)	0 (0.0)	0 (0.0)	0 (0.0)	0 (0.0)	1 (14.3)	0.41	0.87
**BCL2L1°**	2 (4.5)	1 (25.0)	1 (3.8)	0 (0.0)	0 (0.0)	0 (0.0)	0.46	0.87
**MUTYH**	2 (4.5)	1 (25.0)	1 (3.8)	0 (0.0)	0 (0.0)	0 (0.0)	0.46	0.87
**SMARCA4**	2 (4.5)	0 (0.0)	1 (3.8)	1 (25)	0 (0.0)	0 (0.0)	0.46	0.87
**NF1**	4 (9.1)	0 (0.0)	2 (7.7)	1 (25.0)	0 (0.0)	1 (14.3)	0.60	0.98
**CDKN2A^**	14 (31.8)	1 (25.0)	9 (34.6)	1 (25.0)	2 (66.7)	1 (14.3)	0.62	0.98
**CDKN2B°**	14 (31.8)	1 (25.0)	9 (34.6)	1 (25.0)	2 (66.7)	1 (14.3)	0.62	0.98
**ARID1A**	3 (6.8)	0 (0.0)	2 (7.7)	1 (25.0)	0 (0.0)	0 (0.0)	0.63	0.98
**FGFR1^**	3 (6.8)	0 (0.0)	2 (7.7)	1 (25.0)	0 (0.0)	0 (0.0)	0.63	0.98
**GNAS**	3 (6.8)	0 (0.0)	2 (7.7)	1 (25.0)	0 (0.0)	0 (0.0)	0.63	0.98
**NFE2L2**	2 (4.5)	0 (0.0)	1 (3.8)	0 (0.0)	0 (0.0)	1 (14.3)	0.65	0.98
**STK11**	6 (13.6)	0 (0.0)	5 (19.2)	1 (25.0)	0 (0.0)	0 (0.0)	0.68	1.00
**ATM**	5 (11.4)	0 (0.0)	5 (19.3)	0 (0.0)	0 (0.0)	0 (0.0)	0.77	1.00
**RET**	4 (9.1)	0 (0.0)	4 (15.4)	0 (0.0)	0 (0.0)	0 (0.0)	0.86	1.00
**ALK**	1 (2.3)	0 (0.0)	1 (3.8)	0 (0.0)	0 (0.0)	0 (0.0)	1.00	1.00
**APC**	1 (2.3)	0 (0.0)	1 (3.8)	0 (0.0)	0 (0.0)	0 (0.0)	1.00	1.00
**ATR**	1 (2.3)	0 (0.0)	1 (3.8)	0 (0.0)	0 (0.0)	0 (0.0)	1.00	1.00
**CDH1**	1 (2.3)	0 (0.0)	1 (3.8)	0 (0.0)	0 (0.0)	0 (0.0)	1.00	1.00

**CDK8**	1 (2.3)	0 (0.0)	1 (3.8)	0 (0.0)	0 (0.0)	0 (0.0)	1.00	1.00
**ERBB2^**	1 (2.3)	0 (0.0)	1 (3.8)	0 (0.0)	0 (0.0)	0 (0.0)	1.00	1.00
**KDM5C**	1 (2.3)	0 (0.0)	1 (3.8)	0 (0.0)	0 (0.0)	0 (0.0)	1.00	1.00
**KDR^**	1 (2.3)	0 (0.0)	1 (3.8)	0 (0.0)	0 (0.0)	0 (0.0)	1.00	1.00
**KIT^**	3 (6.8)	0 (0.0)	3 (11.5)	0 (0.0)	0 (0.0)	0 (0.0)	1.00	1.00
**MLH1**	2 (4.5)	0 (0.0)	2 (7.7)	0 (0.0)	0 (0.0)	0 (0.0)	1.00	1.00
**MYC**	4 (9.1)	0 (0.0)	3 (11.5)	0 (0.0)	0 (0.0)	1 (14.3)	1.00	1.00
**PDGFRA^**	2 (4.5)	0 (0.0)	2 (7.7)	0 (0.0)	0 (0.0)	0 (0.0)	1.00	1.00
**PRKDC**	1 (2.3)	0 (0.0)	1 (3.8)	0 (0.0)	0 (0.0)	0 (0.0)	1.00	1.00
**SMAD4**	1 (2.3)	0 (0.0)	1 (3.8)	0 (0.0)	0 (0.0)	0 (0.0)	1.00	1.00
**WT1**	1 (2.3)	0 (0.0)	1 (3.8)	0 (0.0)	0 (0.0)	0 (0.0)	1.00	1.00

* p-value based on the Fisher's exact for categorical variables; † Correction for multiple comparisons according to Benjamini–Hochberg. CoADC, combined-LCNEC with adenocarcinoma; CoSQC, combined-LCNEC with squamous cell carcinoma; LCNEC NAP+, LCNECs showing only immunohistochemical napsin-A positivity but no evidence of a distinct conventional ADC pattern; CoSCLC, combined-LCNEC with small cell neuroendocrine carcinoma. ^Mutation and CNV was considered as identical impact. °CNV was considered.
