# Supplementary material for: Combined Large Cell Neuroendocrine Carcinomas of the Lung: Integrative Molecular Analysis Identifies Subtypes with Potential Therapeutic Implications
Source: Cancers (Basel). 2022 Sep 24;14(19):4653. doi: 10.3390/cancers14194653 (PMC9562868; doi:10.3390/cancers14194653)
Supplement: Supplementary file 1 [file cancers-14-04653-s001.zip › Table S9.pdf]

Supplementary Table S9. List of gene set included in Hallmark, C2, C5 and C6 MSigDB and GSEA score for each cluster.

Gene set	CL4	CL7	CL9
msigdb_h_HALLMARK_INFLAMMATORY_RESPONSE	-0.322131387618288	0.0132224090583122	0.135713949308913
msigdb_h_HALLMARK_INTERFERON_GAMMA_RESPONSE	-0.586347357688907	0.111045524420133	0.378015970500968
msigdb_h_HALLMARK_INTERFERON_ALPHA_RESPONSE	-0.729749979964741	-0.1038395828687	0.250726658168237
msigdb_h_HALLMARK_COAGULATION	-0.35949163852475	0.058603496110085	-0.027171044145026
msigdb_h_HALLMARK_E2F_TARGETS	-0.536962435028556	0.0976670634813956	0.466949839549773
msigdb_h_HALLMARK_TNFA_SIGNALING_VIA_NFKB	-0.196813228890088	0.504102780177645	0.700685097944464
msigdb_h_HALLMARK_ALLOGRAFT_REJECTION	-0.238766836674156	0.278257749184253	0.438455698443045
msigdb_h_HALLMARK_G2M_CHECKPOINT	-0.289669997814775	0.299511810808562	0.610959766199573
msigdb_h_HALLMARK_IL6_JAK_STAT3_SIGNALING	-0.295763776833093	0.480275174340868	0.515982848333099
msigdb_h_HALLMARK_COMPLEMENT	-0.569727288054019	0.0963947509986327	0.117191453067071
msigdb_h_HALLMARK_PANCREAS_BETA_CELLS	-0.00108661454676695	-0.0619241125820332	-0.340840030068329
msigdb_h_HALLMARK_EPITHELIAL_MESENCHYMAL_TRANSITION	-0.434740856842385	0.398590328151383	0.711582421187312
msigdb_h_HALLMARK_KRAS_SIGNALING_UP	-0.658087319740875	0.0108113961781402	0.185558718445237
msigdb_h_HALLMARK_ANGIOGENESIS	-0.737821716997291	0.158184592886559	0.359438824983272
msigdb_h_HALLMARK_SPERMATOGENESIS	0.318073297403356	-0.326739379517981	0.180062733789443
msigdb_h_HALLMARK_XENOBIOTIC_METABOLISM	-0.983600047460958	-0.0538515558304031	-0.17960288458154
msigdb_h_HALLMARK_IL2_STAT5_SIGNALING	-0.889989292207312	0.279754713905487	0.484045472389731
msigdb_h_HALLMARK_APOPTOSIS	-1	0.0748282012266754	0.322815733635732
msigdb_h_HALLMARK_FATTY_ACID_METABOLISM	-1	-0.23412373724289	-0.255438250360197
msigdb_h_HALLMARK_REACTIVE_OXYGEN_SPECIES_PATHWAY	-1	0.50886978587198	0.335585144538539
msigdb_h_HALLMARK_P53_PATHWAY	-1	0.0721208492579906	0.165006224826113
msigdb_h_HALLMARK_GLYCOLYSIS	-1	0.022461860857645	-0.0586540732463449
msigdb_h_HALLMARK_HYPOXIA	-1	-0.00542654811501042	0.187747187845311
msigdb_h_HALLMARK_WNT_BETA_CATENIN_SIGNALING	-0.848081287565344	0.00182756979579191	0.182654805065267
msigdb_h_HALLMARK_NOTCH_SIGNALING	-0.423475960508287	0.596598159527687	0.466604108504745
msigdb_h_HALLMARK_APICAL_SURFACE	-0.0426326957851585	0.0306633755272918	0.317538713762216
msigdb_h_HALLMARK_ESTROGEN_RESPONSE_EARLY	-1	0.376019431494749	-0.0502993660564927
msigdb_h_HALLMARK_CHOLESTEROL_HOMEOSTASIS	-1	0.268090530576296	0.333312942008077
msigdb_h_HALLMARK_APICAL_JUNCTION	-1	-0.18137905441456	0.301492206175636
msigdb_h_HALLMARK_MTORC1_SIGNALING	-1	0.427644549684412	0.556276358555538
msigdb_h_HALLMARK_MYC_TARGETS_V2	-1	0.427703380030959	0.456165668631292
msigdb_h_HALLMARK_TGF_BETA_SIGNALING	-1	0.282225969461975	0.42447306708096
msigdb_h_HALLMARK_MYC_TARGETS_V1	-1	0.387063822725721	0.794047342166784
msigdb_h_HALLMARK_OXIDATIVE_PHOSPHORYLATION	-1	0.0799144792135014	0.063846582967821
msigdb_h_HALLMARK_UNFOLDED_PROTEIN_RESPONSE	-1	0.727797822514425	0.767392641914131
msigdb_h_HALLMARK_PROTEIN_SECRETION	-1	0.502124790209172	0.477030865208254
msigdb_h_HALLMARK_MITOTIC_SPINDLE	-1	0.214648322221636	0.59951476243665
msigdb_h_HALLMARK_ANDROGEN_RESPONSE	-1	0.62582773190936	0.739944987460592
msigdb_h_HALLMARK_DNA_REPAIR	-1	0.365232442608628	0.643809880817308
msigdb_h_HALLMARK_UV_RESPONSE_DN	-1	0.293778466037279	0.599991069016861
msigdb_h_HALLMARK_ADIPOGENESIS	-1	0.0131752674182972	0.000384338794624295
msigdb_h_HALLMARK_PEROXISOME	-1	-0.257663585609044	-0.0477053137969529
msigdb_h_HALLMARK_HEDGEHOG_SIGNALING	-0.47332196870072	0.239765612057765	0.378587078196571
msigdb_h_HALLMARK_BILE_ACID_METABOLISM	-1	-0.64824526703479	-0.624036863136721
msigdb_c2_WEBER_METHYLATED_LCP_IN_FIBROBLAST_UP	1	-0.208842888961432	-0.403031411454263
msigdb_c2_WEBER_METHYLATED_LCP_IN_SPERM_UP	1	-0.263999559368973	-0.506493757636425
msigdb_c2_WEBER_METHYLATED_ICP_IN_SPERM_DN	1	-0.206492712862863	0.249317676182301
msigdb_c2_KINNEY_DNMT1_METHYLATION_TARGETS	1	-0.107386536343272	-0.620698564219864
msigdb_c2_WP_TYPE_III_INTERFERON_SIGNALING	-1	0.469151946522196	0.778661874100241
msigdb_c2_WP_NICOTINE_METABOLISM	1	-0.388714552033862	-0.234499181480602
msigdb_c2_WEBER_METHYLATED_HCP_IN_FIBROBLAST_DN	1	-0.19343946167622	-0.0886877846426803
msigdb_c2_WEBER_METHYLATED_HCP_IN_SPERM_DN	1	-0.414056972893734	-0.20126087368725
msigdb_c2_REACTOME_LOSS_OF_FUNCTION_OF_SMAD2_3_IN_CANCER	-1	0.476365445905935	1
msigdb_c2_REACTOME_G2_M_DNA_REPLICATION_CHECKPOINT	-0.403431015727398	0.372462843313471	0.615939450882853
msigdb_c2_WEBER_METHYLATED_LCP_IN_SPERM_DN	1	-0.62905507550551	0.209148430936276
msigdb_c2_WEBER_METHYLATED_ICP_IN_FIBROBLAST	1	-0.601032483401118	0.402576698519131
msigdb_c2_TESAR_ALK_TARGETS_EPISC_3D_UP	0.663067964320905	0.0153901914148762	0.743483308909705
msigdb_c2_TESAR_ALK_AND_JAK_TARGETS_MOUSE_ES_D4_UP	0.135970469045573	-0.156601783958834	0.743343749499684
msigdb_c2_JI_CARCINOGENESIS_BY_KRAS_AND_STK11_UP	-0.906129434442574	-1	-0.101681970914616
msigdb_c2_REACTOME_GRB7_EVENTS_IN_ERBB2_SIGNALING	-1	0.128603728768972	-0.807108186550556
msigdb_c2_WEBER_METHYLATED_LCP_IN_FIBROBLAST_DN	1	-0.645920633475564	-0.0374939188247893

msigdb_c2_MCCOLLUM_GELDANAMYCIN_RESISTANCE_DN	-0.816186958890006	-0.312302173559673	1
msigdb_c2_REACTOME_FGFR2B_LIGAND_BINDING_AND_ACTIVATION	-0.462868895176659	-0.350486972879286	1
msigdb_c2_TESAR_ALK_TARGETS_EPISC_4D_UP	0.269110630975975	0.124119855692457	1
msigdb_c2_BIOCARTA_IFNG_PATHWAY	-0.982542063888494	0.215734810197984	0.917342757647644
msigdb_c2_MIZUKAMI_HYPOXIA_DN	-0.932460183895656	-0.476184557087365	0.311959527366952
msigdb_c2_WP_KIT_RECEPTOR_SIGNALING_PATHWAY	-1	1	0.755408115744033
msigdb_c2_REACTOME_RET_SIGNALING	-1	1	0.575776589066207
msigdb_c2_REACTOME_MET_ACTIVATES_RAS_SIGNALING	-1	1	0.98085654815245
msigdb_c2_REACTOME_ERK_MAPK_TARGETS	-1	1	0.607388152715192
msigdb_c2_REACTOME_MAPK3_ERK1_ACTIVATION	-0.909644710869656	1	0.798303398011746
msigdb_c5_WP_CANCER_IMMUNOTHERAPY_BY_CTLA4_BLOCKADE	-1	0.426801455986178	0.896069702784337
msigdb_c6_HINATA_NFKB_IMMU_INF	-0.379293141575261	0.133667768053069	0.205324354263691
msigdb_c6_SINGH_KRAS_DEPENDENCY_SIGNATURE	-0.40633805170397	0.401615548866986	-0.126756861393776
msigdb_c6_RPS14_DN.V1_UP	-0.44699709317281	0.0460787583464091	0.241400593598091
msigdb_c6_CAHOY_ASTROGLIAL	-0.270530875675306	0.451796111880679	0.40626184670094
msigdb_c6_CAHOY_NEURONAL	-0.0263468517861005	-0.76254538580289	-0.417260977678663
msigdb_c6_EGFR_UP.V1_UP	-0.464479035692183	0.484749041060108	0.518631711968778
msigdb_c6_BMI1_DN.V1_UP	-0.360416399630395	0.154868523262605	0.335365017299661
msigdb_c6_E2F1_UP.V1_DN	-0.621953072813885	0.368535616272335	0.336797736475768
msigdb_c6_HINATA_NFKB_MATRIX	-1	-0.183861149247845	0.449078853415058
msigdb_c6_ESC_J1_UP_LATE.V1_UP	-0.527033893463098	0.0849104000667822	-0.0578939992791977
msigdb_c6_P53_DN.V2_UP	0.432970804966554	0.57134395226153	0.418543572219019
msigdb_c6_HOXA9_DN.V1_UP	-0.863649860188815	0.193227516195769	0.277854221291157
msigdb_c6_E2F3_UP.V1_UP	-0.118039989446532	-0.270711793870835	0.0126773099422031
msigdb_c6_MEL18_DN.V1_UP	-0.536375695369986	0.131943677199498	0.286415756800106
msigdb_c6_ATF2_UP.V1_DN	-0.618934853246494	0.13948679330723	0.242511151199911
msigdb_c6_RPS14_DN.V1_DN	-0.388784036973668	0.27636744375116	0.57087939547084
msigdb_c6_BMI1_DN_MEL18_DN.V1_UP	-0.279922900053739	0.181457755541542	0.431044407984554
msigdb_c6_STK33_NOMO_UP	-0.799685811392795	0.154883129286083	0.316230500556262
msigdb_c6_ATF2_S_UP.V1_DN	-0.30213242834101	0.162578506712798	0.0950957020864116
msigdb_c6_LEF1_UP.V1_DN	-0.795899705823486	0.123744477265821	-0.230985505025669
msigdb_c6_KRAS.KIDNEY_UP.V1_UP	-0.11677933315183	-0.655993057447143	-0.134531573091187
msigdb_c6_RB_DN.V1_DN	-0.918159865186344	0.224029476275889	0.233388338282297
msigdb_c6_KRAS.50_UP.V1_DN	0.539196809989228	-0.601062343580912	0.345052918353314
msigdb_c6_KRAS.LUNG_UP.V1_DN	-0.192866290695735	-0.484426459462283	0.0413008033734654
msigdb_c6_ESC_V6.5_UP_EARLY.V1_DN	-0.487975820185089	0.182970315900215	0.270424644558639
msigdb_c6_MEK_UP.V1_UP	-0.983081660728961	0.57517521401663	0.0767310619649193
msigdb_c6_SIRNA_EIF4GI_UP	-1	-0.0397889327510369	0.119071122607142
msigdb_c6_RB_P107_DN.V1_DN	-1	0.207072393829706	0.326681736146944
msigdb_c6_AKT_UP.V1_UP	-1	0.0725888387827749	-0.091176911680822
msigdb_c6_KRAS.BREAST_UP.V1_DN	1	-0.111974700941868	0.0482691879241577
msigdb_c6_TBK1.DF_UP	-1	0.182960864147855	0.392104106338262
msigdb_c6_CORDENONSI_YAP_CONSERVED_SIGNATURE	-0.586522987006265	0.324528636547843	0.862671906961161
msigdb_c6_PTEN_DN.V2_UP	0.198232063754509	0.146028653187255	0.446325256424226
msigdb_c6_IL2_UP.V1_UP	0.249202195296494	0.563638883635526	0.604684103501734
msigdb_c6_CSR_LATE_UP.V1_UP	-1	-0.0043097649437619	0.813693394568047
msigdb_c6_TBK1.DF_DN	-1	0.504274109872546	0.931469551554599
msigdb_c6_TBK1.DN.48HRS_DN	-1	0.396459883535919	0.691738177405214
msigdb_c6_RAF_UP.V1_UP	-1	0.286859828410703	0.323844471430603
msigdb_c6_KRAS.AMP.LUNG_UP.V1_UP	1	-0.252037271354561	-0.463046857519469
msigdb_c6_GLI1_UP.V1_DN	-1	0.461344520841493	0.0586314463633051
msigdb_c6_CTIP_DN.V1_UP	1	-0.0900917935305577	-0.321458710991931
msigdb_c6_CSR_EARLY_UP.V1_UP	-1	0.71907925520803	0.857239493206572
msigdb_c6_CAMP_UP.V1_UP	-1	0.613335040749133	0.591587463658405
msigdb_c6_GLI1_UP.V1_UP	-1	-0.0261772351337703	0.296514778987925
msigdb_c6_SIRNA_EIF4GI_DN	-1	0.470347195445933	0.759000969878868
msigdb_c6_GCNP_SHH_UP_LATE.V1_UP	-1	0.277769523159385	0.708655330516007
msigdb_c6_HOXA9_DN.V1_DN	-1	0.34496679015062	1
msigdb_c6_RB_P107_DN.V1_UP	-0.741890220315945	-0.0405812943678413	0.229746687088223
msigdb_c6_KRAS.50_UP.V1_UP	-0.764519888485867	-0.526554009527644	-0.443490977332402
msigdb_c6_ALK_DN.V1_UP	0.754198241540285	1	0.651525116963562
msigdb_c6_KRAS.300_UP.V1_UP	-0.0934908618046596	-0.200363118248607	0.0157488706681519
msigdb_c6_ATM_DN.V1_DN	0.214438125299074	-0.00957665433833144	0.0977069825207895

msigdb_c6_RELA_DN.V1_DN	0.471519288126188	0.456016238937981	0.15889303207401
msigdb_c6_SNF5_DN.V1_UP	-0.844642079772407	0.128174221090034	0.628663378941854
msigdb_c6_PRC2_EZH2_UP.V1_DN	-0.487598073999319	-0.0129973302920521	0.394982550166701
msigdb_c6_E2F1_UP.V1_UP	-1	0.344804139038696	0.761564673926349
msigdb_c6_AKT_UP.V1_DN	-0.832610832864926	-0.107589554005867	-0.0445950405115498
msigdb_c6_GCNP_SHH_UP_EARLY.V1_UP	-0.581709877806936	0.342645727737495	0.769279445260881
msigdb_c6_CSR_LATE_UP.V1_DN	-0.745152035230435	-0.259964335381859	-0.178357937687169
msigdb_c6_STK33_UP	-0.680689884186724	0.230702230402788	0.361902513965994
msigdb_c6_RB_DN.V1_UP	-1	0.0196201662852096	-0.117532465163134
msigdb_c6_PRC2_EED_UP.V1_DN	-0.557853223241207	0.215995024092655	0.225410834899449
msigdb_c6_BCAT.100_UP.V1_DN	1	-0.112354420165341	0.173587645478414
msigdb_c6_RB_P130_DN.V1_UP	-1	-0.113221725335911	-0.106637803329515
msigdb_c6_RAPA_EARLY_UP.V1_DN	-1	0.172457176699886	-0.0875473823983023
msigdb_c6_KRAS.600.LUNG.BREAST_UP.V1_UP	0.674202139048552	0.036857329013495	-0.0101628407108637
msigdb_c6_MYC_UP.V1_UP	-1	0.204937617878338	0.327061860221469
msigdb_c6_KRAS.600_UP.V1_UP	0.3466507232276	0.23096604376948	0.32117593724568
msigdb_c6_KRAS.LUNG_UP.V1_UP	0.425407045060222	-0.210122777790076	-0.0318621869425469
