# Supplementary figures and images for: Combined Large Cell Neuroendocrine Carcinomas of the Lung: Integrative Molecular Analysis Identifies Subtypes with Potential Therapeutic Implications
Source: Cancers (Basel). 2022 Sep 24;14(19):4653. doi: 10.3390/cancers14194653 (PMC9562868; doi:10.3390/cancers14194653)

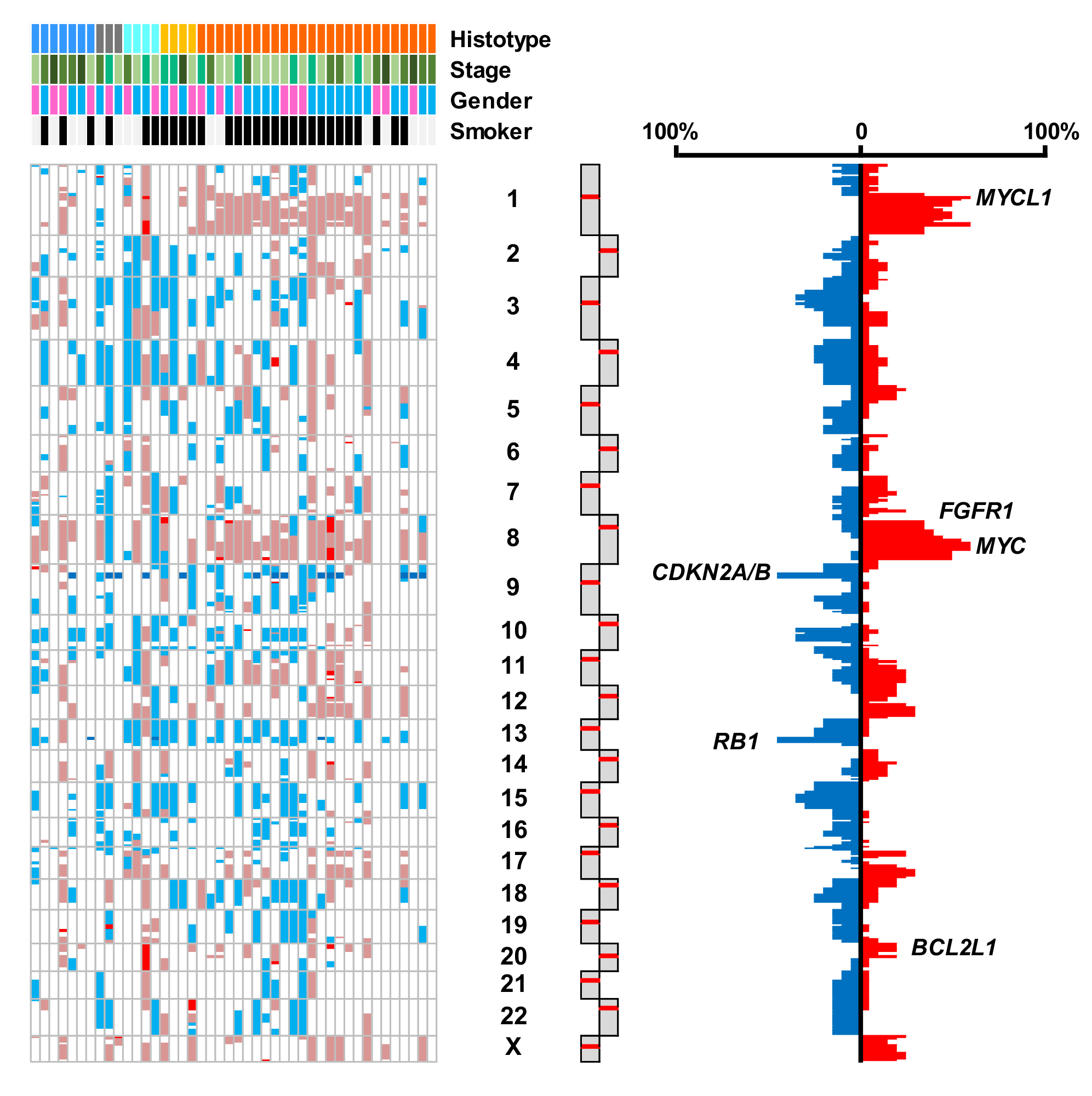

Supplement: Supplementary file 1 [file cancers-14-04653-s001.zip › Figure S1.tif]

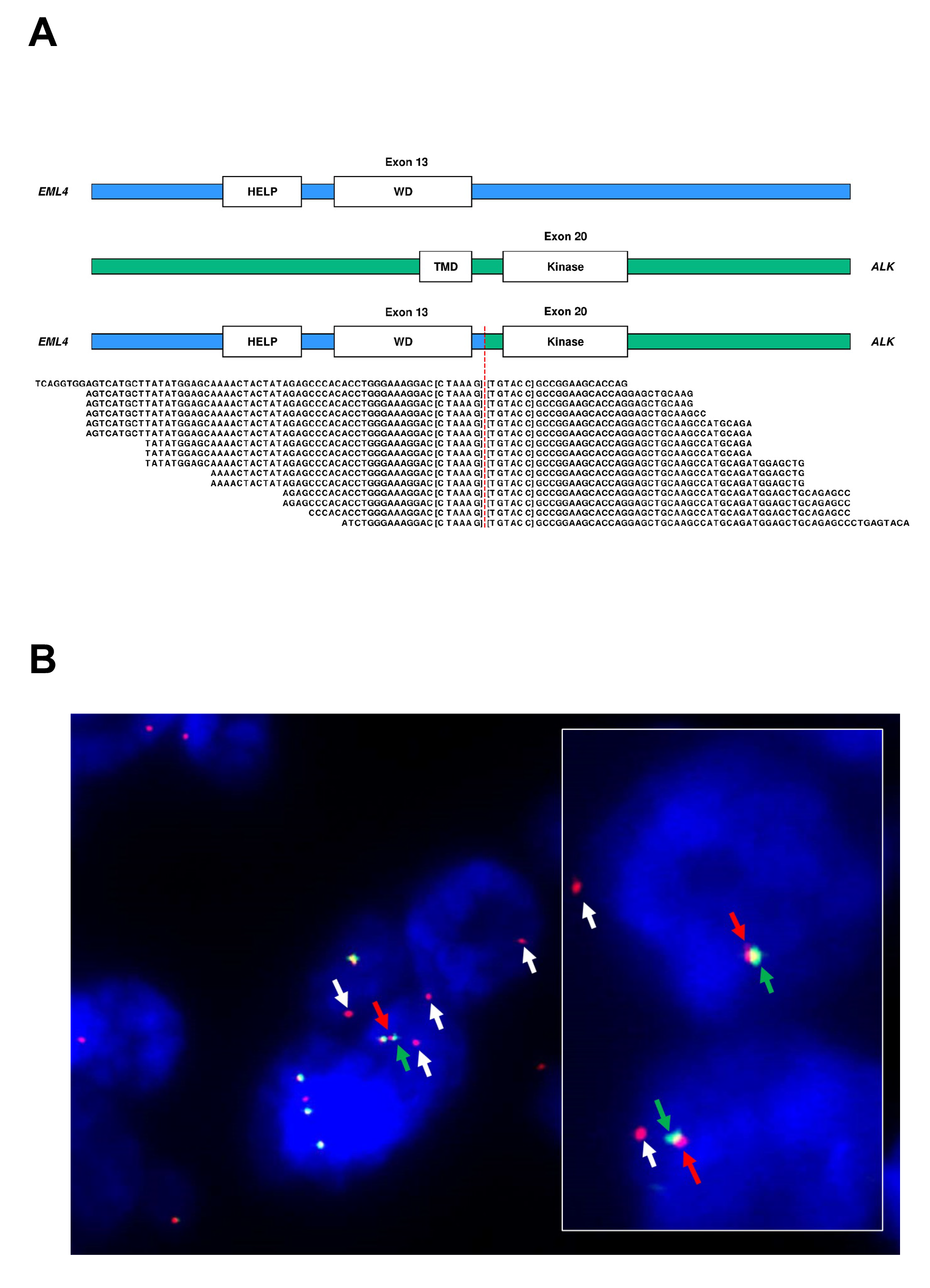

Supplement: Supplementary file 1 [file cancers-14-04653-s001.zip › Figure S2.tif]

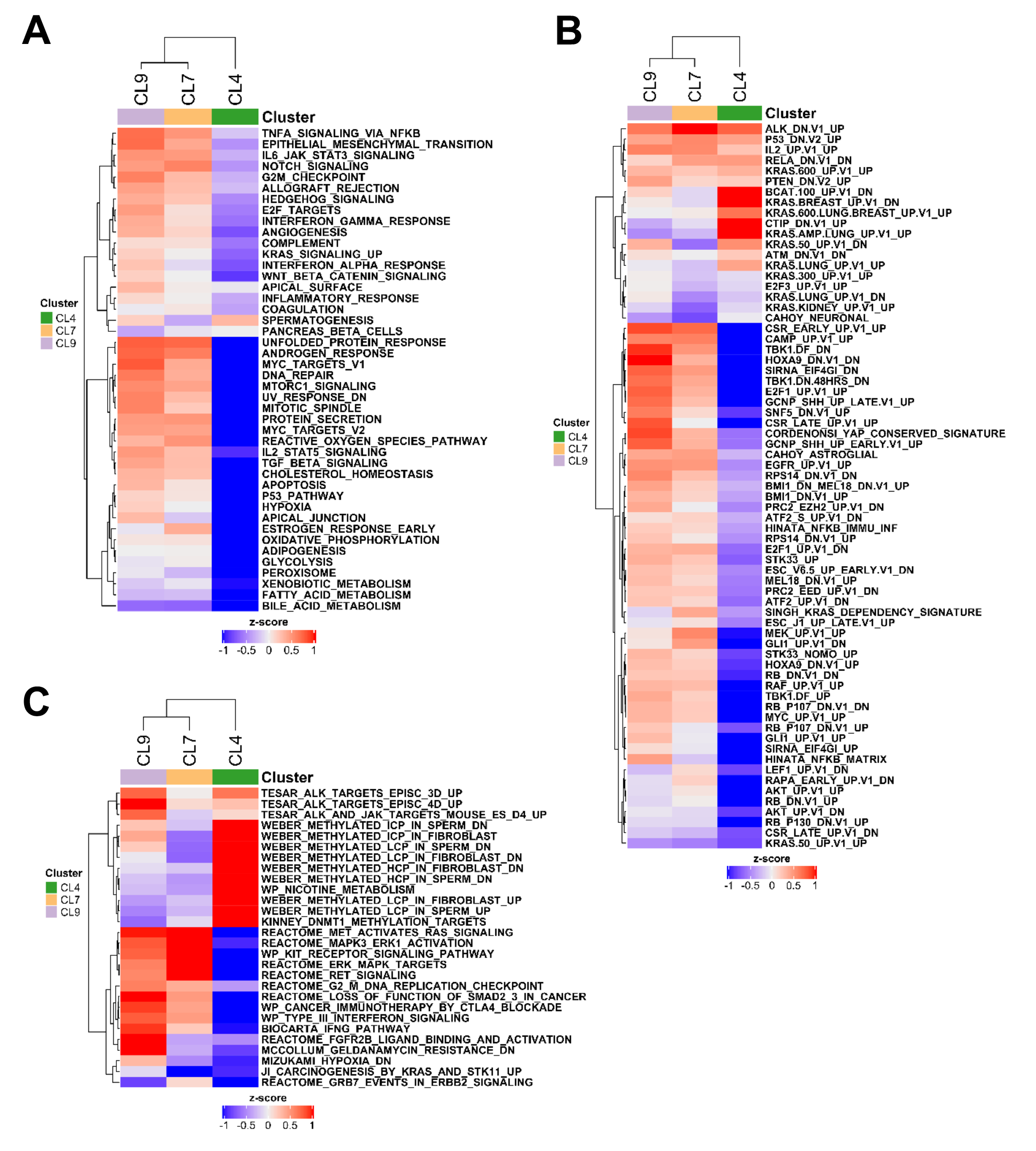

Supplement: Supplementary file 1 [file cancers-14-04653-s001.zip › Figure S3.tif]
